# Aquaporin dysfunction and impaired brain interstitial fluid clearance in Alzheimer’s disease – an integrative review

**DOI:** 10.3389/fncel.2026.1903812

**Published:** 2026-08-18

**Authors:** Marina Daniela Manescu, Teodora Minca, Ionica Pirici, Cristina Jana Busuioc, Valentin Octavian Mateescu, Daniel Pirici

**Affiliations:** 1 Department of Histology, University of Medicine and Pharmacy of Craiova, Craiova, Romania; 2 Department of Human Anatomy, University of Medicine and Pharmacy of Craiova, Craiova, Romania

**Keywords:** Alzheimer’s disease, aquaporin 4, fluid brain clearing, mouse models, paravascular drainage, intramural periarterial drainage

## Abstract

Accumulation of amyloid-beta (Aβ) deposits is one of the neuropathological hallmarks of Alzheimer’s disease (AD), the most frequent neurodegenerative disease in all age groups. While decades of research have focused on the production and aggregation of Aβ peptides, mounting evidence implicate impaired brain fluid dynamics and protein waste clearance as critical contributors to AD pathogenesis. The aquaporin family of water channels, particularly aquaporin-4 (AQP4) and AQP1, has emerged as central regulators of brain interstitial fluid (ISF) homeostasis and Aβ clearance. AQP4, expressed at the perivascular endfeet of astrocytes, is the principal water channel driving fluid convection exchange, acting as a brain-wide network in which ISF and solutes are cleared via venous, paravenous and periarterial routes, as well as through dural lymphatic vessels and along perineural spaces. AQP1, expressed predominantly in the choroid plexus epithelium, governs cerebrospinal fluid (CSF) secretion and thereby modulates the pressure gradients that sustain this convective flow. This review provides an integrated overview of the molecular pathology of AD, the physiological roles of AQP4 and AQP1, together with the major anatomical pathways of ISF and CSF drainage from the brain. We next address to the genetic and pharmacological modulation of AQP4 and AQP1 in transgenic AD mouse models and describe the resulting pathological changes. AQP4 knockout consistently exacerbates Aβ pathology and cognitive deficits, with the abnormal distribution of AQP4 within the astrocyte being sufficient to impair clearance, and these data highlight the critical importance of polarized expression versus bulk expression levels. AQP1 modulation, though less studied, alters CSF dynamics and may influence Aβ clearance indirectly through changes in CSF turnover. Pharmacological agents targeting AQP4 and AQP1 offer promising avenues for therapeutic intervention. Understanding the distinct and intersecting roles of aquaporins in brain fluid homeostasis may yield novel strategies for restoring protein clearance in AD.

## Graphical Abstract

**Figure:**
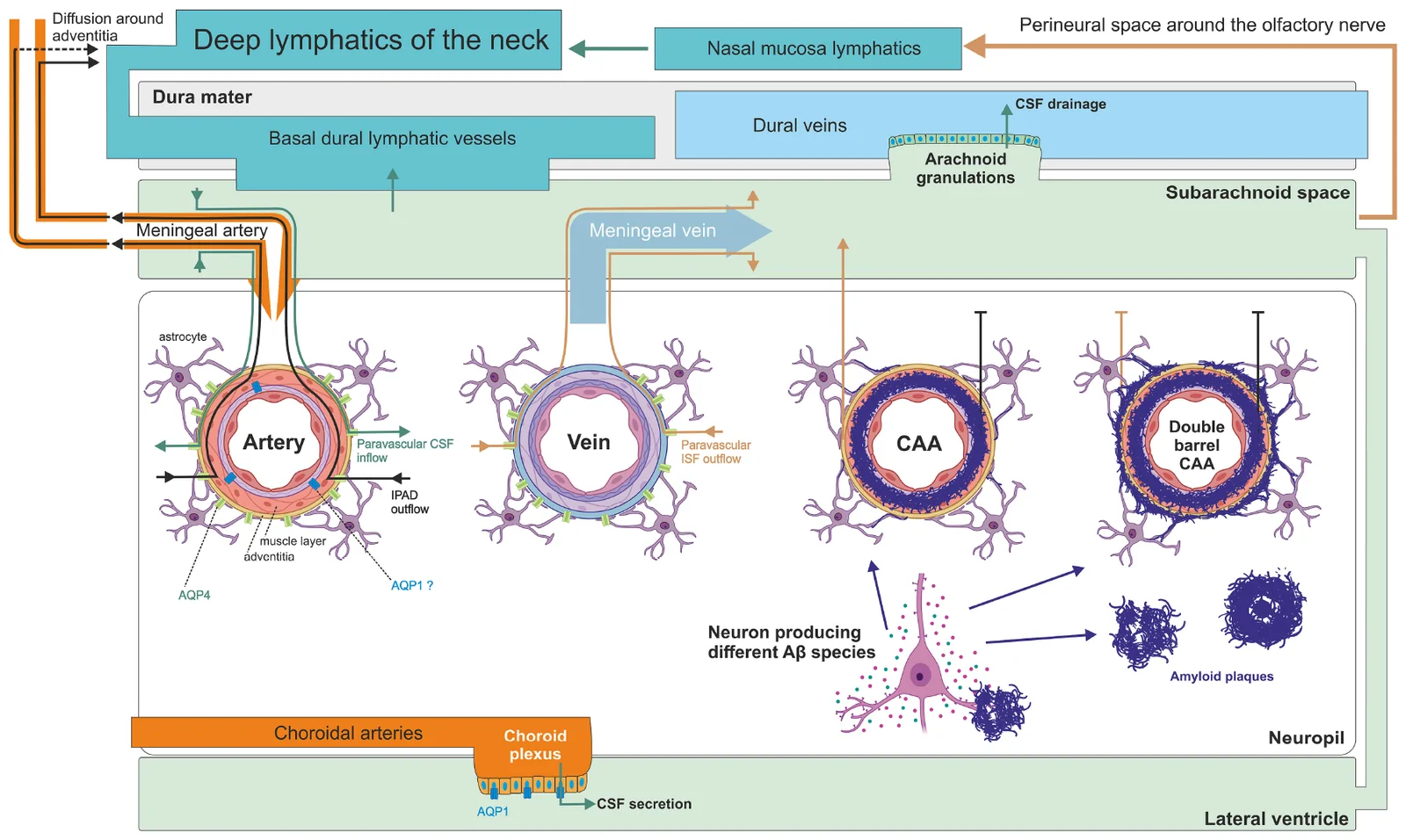
Schematic diagram of water inflow / outflow in the brain. Failure of paravascular outflow and of the IPAD system leads to formation of classical amyloid plaques, CAA and double barrel CAA, when the system is overwhelmed by increased toxic Aβ production (artwork partially created in https://BioRender.com).

## 1 Introduction

Alzheimer’s disease (AD) is the most important neurodegenerative disorder and the leading cause of dementia worldwide, characterized clinically by progressive memory loss and cognitive decline, and pathologically by the accumulation of extracellular amyloid-beta (Aβ) plaques and intracellular neurofibrillary tangles composed of hyperphosphorylated tau protein (Dickson, 2001). The amyloid cascade hypothesis states that the abnormal processing of amyloid precursor protein (APP) generates Aβ peptides species, particularly the aggregation-prone Aβ42 species, which oligomerize and deposit as senile plaques (Hardy and Selkoe, 2002). These aggregates trigger a cascade of neurotoxic events including synaptic dysfunction, tau hyperphosphorylation, neuroinflammation, oxidative stress, and ultimately neuronal loss. Despite decades of research, disease-modifying therapies remain limited, and a growing body of evidence now suggests that impaired clearance of neurotoxic proteins from the neuropil may be an essential contributor to AD pathogenesis. In this context, the water channel proteins aquaporin-4 (AQP4) and AQP1 have emerged as central players in the mechanisms governing brain fluid dynamics and protein clearance, making them compelling targets for further investigation (Badaut et al., 2002; Carbrey and Agre, 2009).

The clearance of brain interstitial fluid (ISF) and the solutes it carries, including Aβ, relies on a complex network of drainage pathways that have gained increasing attention over the past decade, with many complementary routes being described (Iliff et al., 2012). First, the traditional view holds that cerebrospinal fluid (CSF) is reabsorbed into the blood through arachnoid granulations projecting into the dural venous sinuses, though recent evidence suggests this pathway may contribute less to bulk clearance than once believed (Pollay, 2010; Ma et al., 2017). Second, the discovery of a functional meningeal lymphatic system lining the dural sinuses has revolutionized our understanding of brain fluid dynamics; these vessels drain CSF and immune cells toward the deep cervical lymph nodes, and their dysfunction has been directly implicated in age-related cognitive decline and Aβ accumulation in both animal models and human AD (Jaffe et al., 2019). Third, the perivascular and paravascular pathways, or the so called “glymphatic system”, describe a brain-wide network in which CSF enters the brain parenchyma along paraarterial spaces, exchanges with ISF via astrocyte-mediated water flux, and clears solutes along paravenous drainage routes, as well as within the network of basement membranes within the tunica media of arteries (Iliff et al., 2012). Fourth, a major extracranial drainage route also seems to exist along the perineural space of the olfactory nerve, traversing the cribriform plate to reach nasal lymphatics and ultimately the cervical lymph nodes (Chae et al., 2024). This pathway may account for a substantial proportion of CSF efflux and is particularly relevant given the early involvement of the olfactory system in AD pathology.

Central to glymphatic function is AQP4, the predominant water channel in the central nervous system (CNS). AQP4 is expressed primarily on the perivascular endfeet of astrocytes, where it facilitates bidirectional water transport in response to osmotic gradients (Nagelhus and Ottersen, 2013). This highly polarized expression at the level of astrocytes’ endfeets within the perivascular and subpial glia limitans is essential for driving the convective flow of CSF into the brain parenchyma and the subsequent clearance of ISF and interstitial solutes including Aβ. In mice lacking AQP4, glymphatic transport is reduced by approximately 70% and the clearance of exogenously injected Aβ is severely impaired (Iliff et al., 2012). Beyond its role in bulk fluid transport, AQP4 also contributes to extracellular space volume regulation, potassium buffering, synaptic plasticity, and neuroinflammatory responses (Nagelhus and Ottersen, 2013). In the context of AD, the perivascular polarization of AQP4 is disrupted both in human patients and in animal models, with reduced AQP4 coverage of cerebral blood vessels correlating with increased Aβ plaque burden and cognitive decline (Kecheliev et al., 2023).

AQP1 plays a distinct but potentially complementary role in brain fluid homeostasis. Unlike AQP4, AQP1 in the CNS is expressed predominantly in the apical membrane of choroid plexus epithelial cells, where it facilitates CSF secretion by providing the water permeability necessary for transcellular fluid movement, with low-level AQP1 expression being reported in reactive astrocytes and in some neuronal populations under pathological conditions (Tait et al., 2008). The physiological role of AQP1 in CSF production has been convincingly demonstrated in AQP1 knockout mice, which exhibit a 20–25% reduction in CSF secretion rate and reduced intracranial pressure (Oshio et al., 2005). This positions AQP1 as a potential modulator of CSF turnover – a parameter that is intimately linked to glymphatic clearance efficiency, as glymphatic influx is driven in part by the pressure gradients generated by CSF production itself. AQP1 has also been identified in reactive astrocytes surrounding Aβ plaques in both human AD tissue and transgenic mouse models, where it may participate in glial scar formation and local water homeostasis, though its functional significance in this context remains to be fully elucidated (Yamada, 2023).

The objective of this review is to present an integrative view on the physiological roles of aquaporins in the brain in the context of known fluid clearing pathways, pathological implications in AD, and further on to develop on potential therapeutic avenues involving the two most abundant aquaporins in the CNS.

## 2 Molecular pathology of Alzheimer’s disease

The diagnosis of AD is based on exclusion, as several clinical symptoms overlap with those of other neurodegenerative disorders, including frontotemporal dementia and Parkinson’s disease. Despite the development of neurological examinations and brain imaging techniques such as computed tomography (CT) and magnetic resonance imaging (MRI), definitive diagnosis still relies on postmortem brain examination.

The three microscopic pathological hallmarks that characterize AD are neuronal and synaptic loss, extracellular accumulation of amyloid plaques and intracellular neurofibrillary tangles (NFTs) in the cerebral cortex, hippocampus, and amygdala. Among these pathological features, amyloid plaques are thought to represent a central factor capable of triggering hyperphosphorylation of the tau cytoskeletal protein into NFT and subsequent neuronal loss. Consequently, the mechanisms that drive plaque formation have received considerable attention in AD pathogenesis and form the basis of the “amyloid cascade hypothesis”.

**FIGURE 1:**
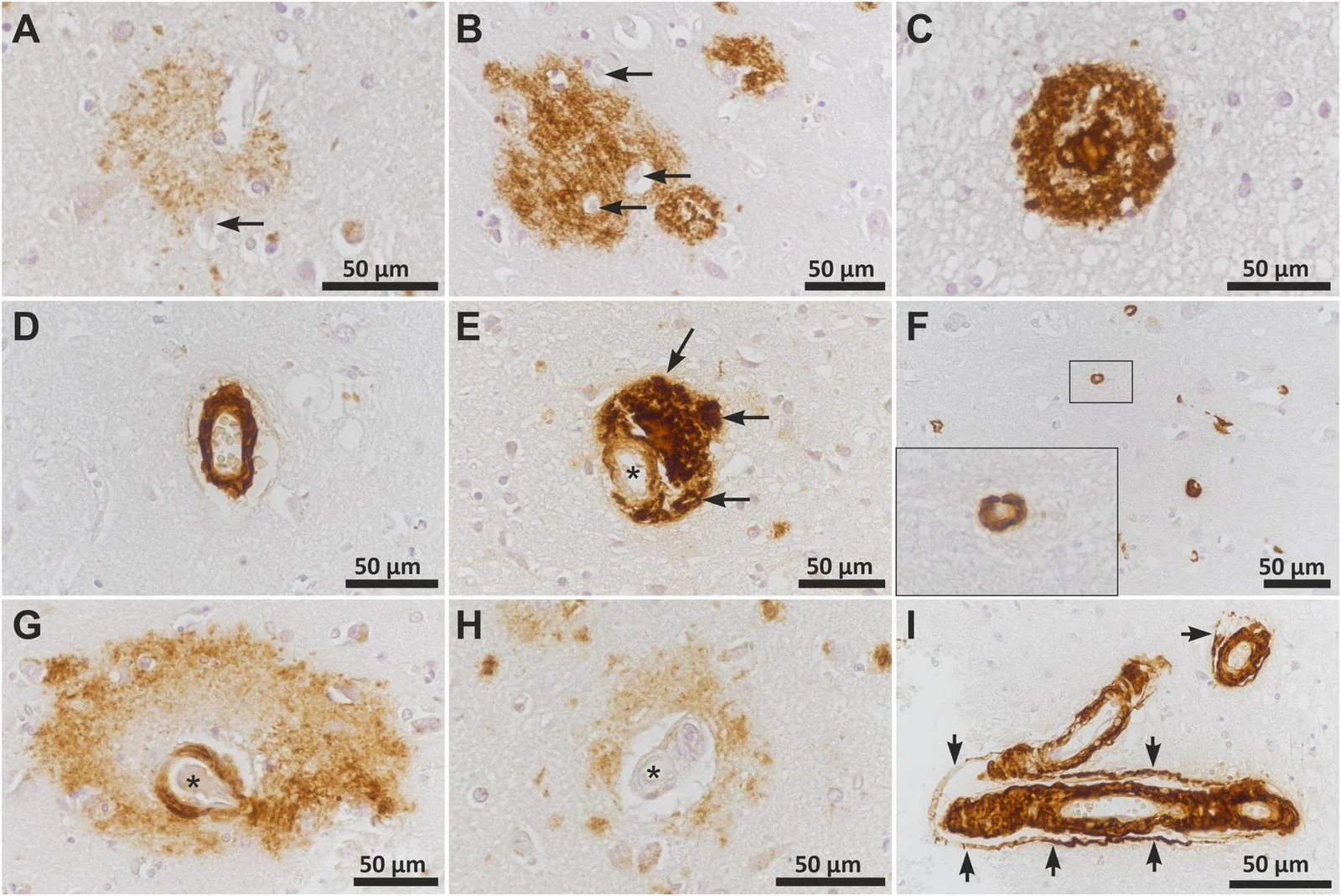
Multiple patterns of amyloid depositions. Exemplary images of different types of amyloid depositions in patients with sporadic senile AD, as detected by immunohistochemistry, such as: **(A)** dense diffuse, **(B)** reticular (build-up around small blood vessels – arrows), and **(C)** dense-core plaques, as well as **(D)** small artery CAA, **(E)** dysphoric angiopathy which contains a vessel with CAA (*) spilling dense material within the neuropil (arrows), **(F)** capillary CAA, **(G)** CAA (*) with dense-diffuse material in the surrounding glia limitans, **(H)** central vessel with no amyloid deposition (*) but with diffuse material in the glia limitans, and **(I)** massive CAA completely replacing the arterial walls, and surrounded by dense accumulations in the detached basement membranes of the glia limitans (arrows) due to dilated para-arterial spaces (the so called “double barrel” appearance). Immunohistochemistry for total Aβ (4G8 antibody).

Senile plaques are extracellular insoluble protein aggregates composed of Aβ, a 40–43 amino acids peptide that migrates as 4 kDa band on SDS gel electrophoresis, and which represents the cleavage product of the ubiquitously expressed APP transmembrane protein. There are many types of amyloid deposits observed in these patients, such as diffuse plaques, dense-core plaques, dense-diffuse plaques, vascular intramural accumulations – cerebral amyloid angiopathy (CAA), or even plaque-CAA complexes – dysphoric angiopathy (Pirici et al., 2011) (Figure 1). Interestingly, the dense-core plaques are mainly composed of 40 amino acids long Aβ (Aβ40), with co-aggregation of 42 amino acid long Aβ (Aβ42), while in contrast, the diffuse plaques are mostly composed of non-fibrillary Aβ42 with less amounts of Aβ40 (Hardy and Selkoe, 2002).

**FIGURE 2:**
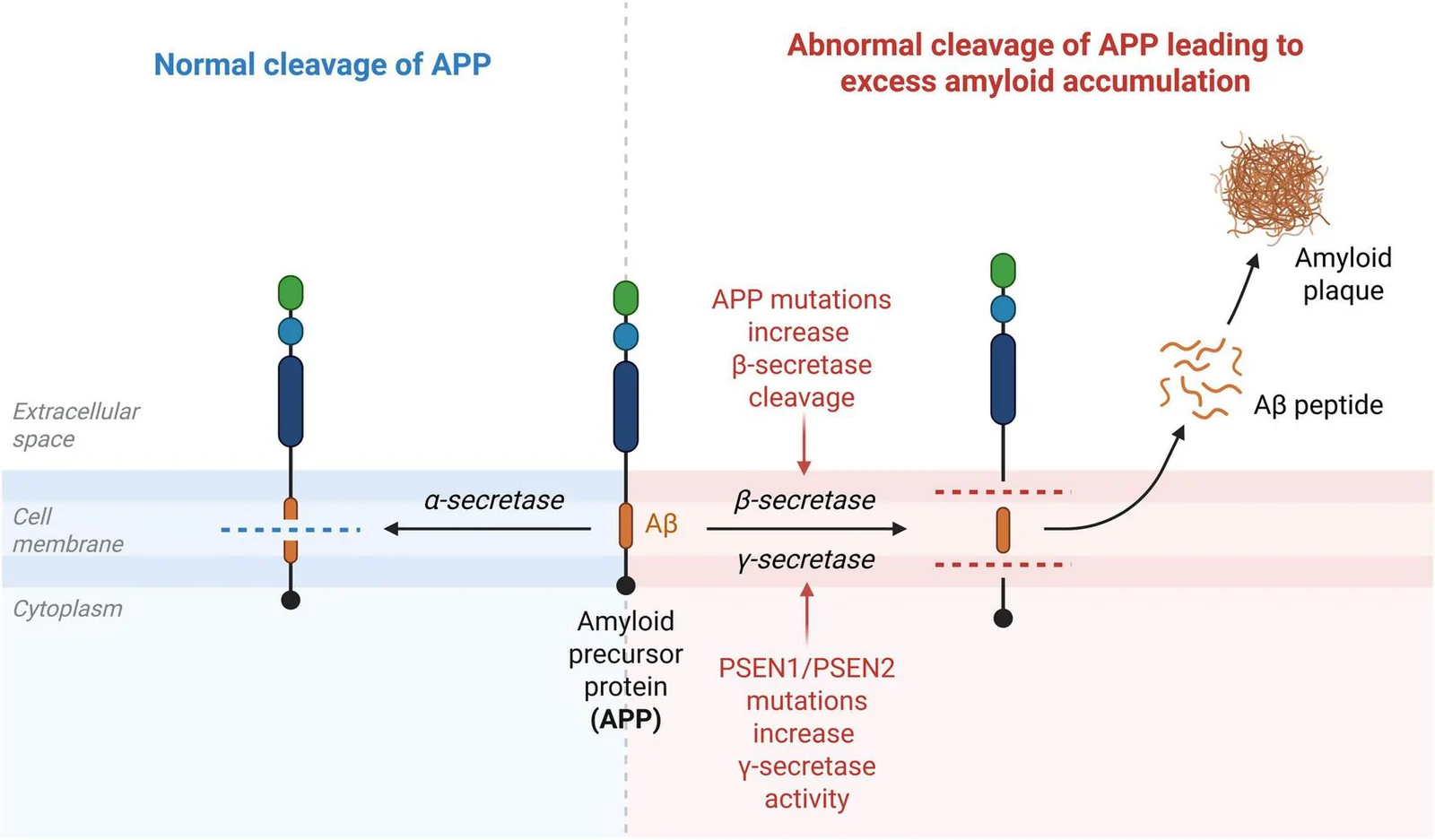
APP processing pathway. APP is a constitutive transmembrane protein which is processed either through the constitutive pathway by the α-secretase resulting in non-toxic fragments, or through the alternative pathway mediated by β and γ secretases, which leads to the formation of the fibrillogenic Aβ that aggregates as insoluble fibrils and amyloid plaques (artwork created in https://BioRender.com).

While age represents the major risk factor for AD, family aggregation, especially before 65 years of age (pre-senile casuistry), has revealed causative mutations in APP, presenilin 1 (PSEN1), and presenilin 2 (PSEN2) genes. These mutations have been shown to promote abnormal cleavage of APP and increased production of Aβ peptide to varying extents. During the constitutive (non-amyloidogenic) processing, APP is first cleaved by an enzyme complex called α-secretase within the Aβ region, and subsequently cut by γ-secretase, generating a short, non-amyloidogenic, p3 peptide. In contrast, during the amyloidogenic processing (according to the so called “amyloid cascade hypothesis”), APP is first cleaved by β-secretase (BACE1) at the N-terminus of the Aβ sequence, followed by the γ-secretase cleavage, producing the aggregation-prone Aβ 40/42 peptides (Figure 2).

Elimination of Aβ from the CNS relies on a network of distinct processes, several of which degrade with aging and during neurodegenerative pathologies. These include: (i) glial cell phagocytosis, as both astrocytes and microglia actively internalize and process extracellular Aβ, (ii) proteolytic degradation by specific enzymes, most notably insulin-degrading enzyme and neprilysin, which chemically break down the peptide, (iii) efflux through the blood-brain barrier (BBB) through low-density lipoprotein receptor-related protein 1 (LRP1) which facilitate the direct transport of Aβ out of the brain tissue and into systemic circulation, and (iv) perivascular/paravascular flow as an exchange between CSF and ISF, which drives waste solutes along these pathways, ultimately routing them to extracranial lymph nodes for drainage (Bonnar et al., 2025). Current neurobiological research focuses heavily on quantifying the exact contribution of each system and on characterizing how these processes alter during illness, and compelling data links perivascular drainage dysfunction directly to the development of AD and CAA.

## 3 Aquaporins in the CNS: an overview

Aquaporins constitute a conserved family of transmembrane channel proteins present across all domains of life, from prokaryotic organisms to mammals, underlining their fundamental biological roles (Engel et al., 2000). In humans, this protein group mediates bidirectional passage of water and, in selected members, additional small solutes across lipid bilayers, enabling precise regulation of cellular and tissue fluid balance. Thirteen aquaporin members aquaporin-0 – aquaporin-12 (AQP0–AQP12) have been identified in human tissues and are categorized according to pore selectivity into three functional classes: (i) water-selective aquaporins, AQP0, AQP1, AQP2, AQP4, AQP5, AQP6 and AQP8, (ii) aquaglyceroporins, AQP3, AQP7, AQP9 and AQP10; and (iii) atypical aquaporins, AQP11 and AQP12, with intracellular localization and with divergent structural and permeability features (Buffoli, 2010). This functional heterogeneity enables aquaporins to participate in diverse physiological processes and contributes to their involvement in multiple pathological states, including neoplastic, renal, neurological, dermatological, metabolic, and cardiovascular disorders. Aquaporins assemble as integral membrane channels that enable rapid trans-bilayer movement of water while maintaining strict exclusion of protons, thereby preserving electrochemical gradients, an essential property for maintaining cellular osmotic stability (Fu and Lu, 2007). As aquaporins display differential substrate selectivity, restricting passage to water molecules, or small neutral solutes such as glycerol, these functional distinctions arise from conserved structural motifs within the channel pore that govern substrate size discrimination, hydrogen bonding, and electrostatic barriers, providing a molecular basis for selective permeability regulation across aquaporin isoforms (De Leon et al., 1991). Aquaporins facilitate rapid osmotic water transport across plasma membranes, enabling efficient near iso-osmolar fluid movement required for transepithelial absorption and secretion in diverse tissues (Verkman, 2002). At the cellular scale, aquaporin-dependent water flux supports dynamic changes in cell volume that are essential for processes such as migration, shape remodeling, and electrically active signaling, including neuroexcitation (Verkman, 2012). In addition to water permeability, aquaglyceroporins mediate glycerol transport, thereby influencing cellular energy balance, lipid metabolism, and proliferative capacity in metabolically active cells (Verkman, 2012).

Multiple members of the aquaporin family are expressed in the adult CNS, with a distribution pattern that reflects specialization across fluid-regulating interfaces. AQP4 represents the most abundantly expressed water channel in the brain parenchyma and is predominantly detected in astrocytes and ependymal cells lining CSF–containing compartments (Lehmann et al., 2004; Papadopoulos and Verkman, 2013). AQP1 expression is classically described as being largely confined to the apical membrane of choroid plexus epithelial cells, consistent with its restricted role at the ventricular CSF interface (Lehmann et al., 2004; Adiele et al., 2025). AQP9 exhibits limited and region-specific expression within the CNS, with reported localization in select glial populations and the choroid plexus, although its overall abundance remains low compared with AQP4 and AQP1 (Papadopoulos and Verkman, 2013; Adiele et al., 2025). Additional family members, including AQP3, AQP5, and AQP8, have been detected at the transcript level in cultured astrocytes, but their protein expression and functional relevance *in vivo* appear minimal under physiological conditions (Lehmann et al., 2004). In recent years, another aquaporin, AQP11 has been found to be expressed in the brain, although at lower levels than those observed in the kidney and thymus (Koike et al., 2016). While AQP4 is a classic plasma-membrane channel, AQP11 is localized on the membranes of the endoplasmic reticulum where it channels hydrogen peroxide bidirectionally to control oxidative stress, and has been identified in the choroid plexus epithelium and brain capillary endothelium, as well as in neurons and pericytes, suggesting also a role in trans-cellular water transport. In AQP11-deficient mice, AQP4 expression was reduced by half, indicating a possible interaction between AQP11 and AQP4 in what it regards fluid metabolism in the CNS (Koike et al., 2016). It is conceivable that loss of AQP11 not only blocks water passage across the endothelial cell layer, but also shifts the local trans-cellular oxidative signaling, to which astrocytes respond by drastically downregulating their own membrane AQP4 expression (Amro et al., 2024).

## 4 Physiology of AQP4 in the CNS

AQP4 exhibits a highly polarized distribution within the cellular compartments in the adult CNS, being predominantly expressed in astrocytic plasma membranes that interface with the BBB (Papadopoulos and Verkman, 2013). During development, AQP4 is weakly detected at the age of 16-22 weeks antepartum, but it shows its mature expression pattern in the perivascular astrocyte endfeets starting with the weeks 24–28, and contributing to the maturation and osmotic regulation around the BBB (Gomori et al., 2006). At the subcellular level, adult life AQP4 is specifically enriched in astrocytic endfeet surrounding cerebral microvessels and along subpial glia limitans, whereas lower densities are detected in non-endfeet astrocytic domains (Nagelhus et al., 2004). This spatial segregation mirrors the organization of perivascular and CSF interfaces, indicating that AQP4 polarization is a defining feature of astrocytic specialization (Lehmann et al., 2004). Maintenance of polarized AQP4 localization depends on its structural anchoring mechanisms, involving dystrophin-associated protein complexes including α-syntrophin, and extracellular matrix components such as agrin, which collectively stabilize channel assemblies at perivascular membranes (Nagelhus et al., 2004; Papadopoulos and Verkman, 2013). In addition, AQP4 is organized into orthogonal arrays of particles within the astrocyte membrane, a supramolecular arrangement that facilitates coordinated tethering of channel clusters rather than individual tetramers (Papadopoulos and Verkman, 2013). Disruption of astrocyte–endothelial or astrocyte–pial contacts leads to loss of AQP4 polarization, underscoring the requirement for intact cellular interfaces in preserving its compartmentalized distribution within the CNS (Papadopoulos and Verkman, 2013). Through its polarized astroglial distribution, AQP4 is functionally coupled to potassium buffering mechanisms, enabling activity-dependent clearance of extracellular K^+^ and stabilization of neuronal excitability during synaptic transmission (Amiry-Moghaddam et al., 2004a; Assentoft et al., 2015). Experimental evidence further indicates that AQP4-mediated water permeability participates in astrocyte migration and morphological plasticity, processes that depend on localized cell volume changes rather than active ion transport (Iacovetta et al., 2012). Collectively, these physiological functions position AQP4 as a key astrocytic determinant of water–ion coupling and microenvironmental homeostasis in the healthy CNS (Nagelhus and Ottersen, 2013).

A plethora of pathological conditions of the CNS associate with AQP4-mediated water imbalance and buildup of cerebral edema, such as ischemic/hemorrhagic stroke, traumatic brain injury (TBI), brain tumors (gliomas), meningitis/abscesses, hydrocephalus, and overall astrocyte loss in neuromyelitis optica, with published data promoting AQP4 inhibition as potent tool for mitigating extracellular fluid accumulations (Nagelhus and Ottersen, 2013; Pirici et al., 2017; Catalin et al., 2018). Important, aquaporin dysregulation extends beyond changes in total protein expression and is more accurately characterized by alterations in subcellular distribution and loss of polarized membrane localization (Amiry-Moghaddam et al., 2004a). Experimental studies across diverse CNS injury and disease models have demonstrated a consistent redistribution of AQP4 away from perivascular astrocyte endfeet toward non-specialized astrocytic membrane domains, indicating that depolarization represents a unifying mechanistic feature of AQP4 dysfunction (Mogoanta et al., 2014; Assentoft et al., 2015). This spatial reorganization of AQP4 disrupts coordinated astrocyte–vascular interactions and modifies local water flux dynamics, thereby altering extracellular microenvironmental stability independently of channel expression levels (Vandebroek and Yasui, 2020).

AQP4 expression and distribution in the astrocytes appear to change differently during normal aging and early AD-related pathology. Thus, in non-dementia aging brains, its immunoreactivity tends to increase in both the grey and white matters, often acquiring a more granular pattern and showing a stronger association with vascular profiles, suggesting an age-related adaptation of astrocytic water-handling mechanisms (Mogoanta et al., 2014; Owasil et al., 2020). In contrast, early AD-associated vascular pathology is characterized by a further increased expression in the grey matter, with loss of endfeet polarization and expression across the entire astrocytic cell body and its processes, therefore less centered on the BBB and perivascular glia limitans (Figure 4), and a white matter attenuated-punctate expression patterns. Thus, as pathology progresses toward severe CAA or white matter hyperintensities, AQP4 immunoreactivity decreases significantly despite increased astrocyte reactivity and gliosis, suggesting that reactive AD-associated astrocytes might in fact gradually lose their ability to buffer water around the BBB (Owasil et al., 2020).

## 5 Physiology of AQP1 in the CNS

AQP1 is a constitutively expressed water channel with a highly restricted but functionally strategic distribution in the CNS. In the adult mammalian brain, AQP1 is predominantly localized to choroid plexus epithelial cells lining the ventricular system, where it is densely expressed at the apical membrane and directly participates in CSF secretion (Yool, 2007; Tait et al., 2008). This spatial confinement distinguishes AQP1 from astrocytic AQP4 and positions it as a regulator of CSF production rather than parenchymal water exchange. During development, AQP1 is detected around weeks 5−7 of embryonic development, on the apical membrane of the primitive epithelial cells forming the choroid plexus, and is active well before the brain is fully structured in order to drive the rapid fluid secretion needed to inflate the embryonic ventricles and generate the early hydrostatic pressure required for normal forebrain differentiation (Gomori et al., 2006).

Mechanistically, AQP1 facilitates rapid osmotic water movement across the choroid plexus epithelium, coupling ion transport to fluid secretion and thereby contributing to the regulation of intracranial fluid homeostasis (Badaut et al., 2002; Tait et al., 2008). Beyond its classical role as a water pore, AQP1 has been identified as a multifunctional component of signaling assemblies, integrating osmotic transport with intracellular regulatory pathways (Yool, 2007). AQP1 function is dynamically modulated by post-translational and signaling mechanisms rather than static expression alone. Phosphorylation-dependent regulation has been demonstrated to influence AQP1 trafficking and membrane insertion, thereby adjusting channel availability at the epithelial surface (Gunnarson et al., 2004). In addition, AQP1 expression is sensitive to extracellular osmolality and ubiquitination pathways, linking environmental cues to channel turnover and functional adaptation (Gunnarson et al., 2004). Notably, in contrast to AQP4, AQP1 water permeability is susceptible to mercury-mediated inhibition and modulation by intracellular signaling cascades, underscoring isoform-specific regulatory properties within the aquaporin family (Gunnarson et al., 2004). Hormonal influences, including steroid hormone signaling, have also been implicated in regulating AQP1 expression, suggesting endocrine control of CSF dynamics under physiological and pathological conditions (Gunnarson et al., 2004). In addition to its role as a water-selective channel, AQP1 also functions as a cyclic guanosine monophosphate (cGMP)-gated cation channel, that it confers ion channel functionality under permissive conditions. In primary epithelial cultures from rat choroid plexus, stimulation with atrial natriuretic peptide (ANP) activates cGMP second-messenger signaling and triggers an AQP1-dependent ionic current. The disappearance of this current after selective AQP1 silencing, without significant changes in background potassium or chloride conductances, supports the specificity of this mechanism for chloride ions (Boassa et al., 2006). Functionally, activation of the AQP1-associated cation conductance decreases fluid movement across polarized choroid plexus epithelium, whereas channel blockade restores transepithelial fluid transport, indicating that this ionic function contributes to the ANP/cGMP-dependent downregulation of CSF secretion (Boassa et al., 2006). Although this mechanism has not been directly tested in relationship to Aβ clearance, CSF production itself helps maintaining the hydrodynamic conditions required for ventricular, subarachnoid, and paravascular fluid movement. Therefore, altered AQP1 ion channel activity could indirectly influence ISF and Aβ clearance by modifying CSF dynamics, CSF–ISF exchange, and finally, the perivascular mobilization of soluble waste products.

Alterations in AQP1 expression and localization have been also implicated in many neurological disorders characterized by disturbed fluid homeostasis. In experimental hydrocephalus, AQP1 expression in the choroid plexus exhibits a biphasic response, with early downregulation followed by marked upregulation at later stages, suggesting a compensatory adaptation aimed at restoring CSF balance (Jeon et al., 2017). This temporal modulation contrasts with parenchymal AQP4 changes and highlights a distinct regulatory role for AQP1 in ventricular fluid disorders. In human intractable epilepsy, AQP1 is aberrantly upregulated in astrocytes within the temporal neocortex, particularly at perivascular glial processes, despite its minimal expression in astrocytes under physiological conditions (Zhou et al., 2008). This ectopic localization implies a pathological redistribution of AQP1 that may contribute to altered water and ion homeostasis in epileptogenic tissue, although causality remains unresolved (Zhou et al., 2008). Functionally, AQP1 overexpression promotes cellular swelling, whereas AQP1 knockdown confers resistance to hypoxia-induced edema, identifying AQP1 as a mechanistic driver of water accumulation under hypoxic stress (Zhang et al., 2013). In the absence of a neurodegenerative disease, physiological AQP1 transcript and protein expression decrease with advancing age, directly impairing CSF production and overall water homeostasis (Stayer-Wilburn et al., 2025). In early-stage AD (Braak and Braak stage II), a significant upregulation of AQP1 has been demonstrated in astrocytes in the vicinity of amyloid plaques in the cerebral cortex, probably in an attempt to handle early neuroinflammation and osmotic imbalance (Hoshi et al., 2012). Surprisingly however, this increase does not persist in advanced AD (Braak and Braak stages V-VI), as total cortical AQP1 levels show large individual variation, resulting in no statistically significant differences from age-matched healthy controls (Perez et al., 2007). It may be that following a persistent state of chronic neuroinflammation and metabolic injuries, after prolonged exposure to toxic amyloid and tau species, these hyper-reactive astrocytes lose their functionality and became an inert glial scar.

Moreover, in adult rodents, AQP1 immunoreactivity has been documented in the smooth muscle cell layer of penetrating arterioles and in the endothelia of capillaries and venules restricted to the subarachnoid space (SAS) vasculature, while being absent in meningeal arterial endothelial cells (Li et al., 2020). However, to date, there is no clear, direct anatomical study demonstrating the same AQP1 localization in the tunica media of human leptomeningeal or penetrating cerebral arteries, therefore the human relevance of this localization remains unconfirmed (Adiele et al., 2025). It is of interest, however, that human studies have not been extensive in this direction, and with no simultaneous immunophenotyping of intra-cellular versus extracellular AQP1 epitopes. As extra-choroidal source of CSF has been previously postulated to correspond to influx of fluid across the BBB, and some CSF production has been suggested to be mediated by endothelial cells, the possible human implications still need to be further explored, including for the presence of region-specific AQP1 isoforms (Reese and Karnovsky, 1967; Li et al., 2020). These observations provide proof-of-concept evidence that AQP1 inhibition may represent a therapeutic strategy for edema associated with nerve injury and ischemic conditions.

## 6 Aquaporins role in the drainage of ISF, as amyloid clearance mechanisms

The brain was long thought to lack conventional lymphatic drainage, yet it still must clear interstitial solutes, excess fluid, and metabolic waste. Many closely related pathways have emerged as central to this process: the paravascular and perivascular spaces, the venous and meningeal lymphatic vessels, as well as perineural drainage pathways. Together, these routes support fluid exchange between CSF, ISF, and peripheral lymphatic circulation. Their dysfunction has been implicated in aging, sleep disruption, traumatic brain injury, stroke, and neurodegenerative diseases such as AD.

### 6.1 Paravascular and perivascular spaces

**FIGURE 3:**
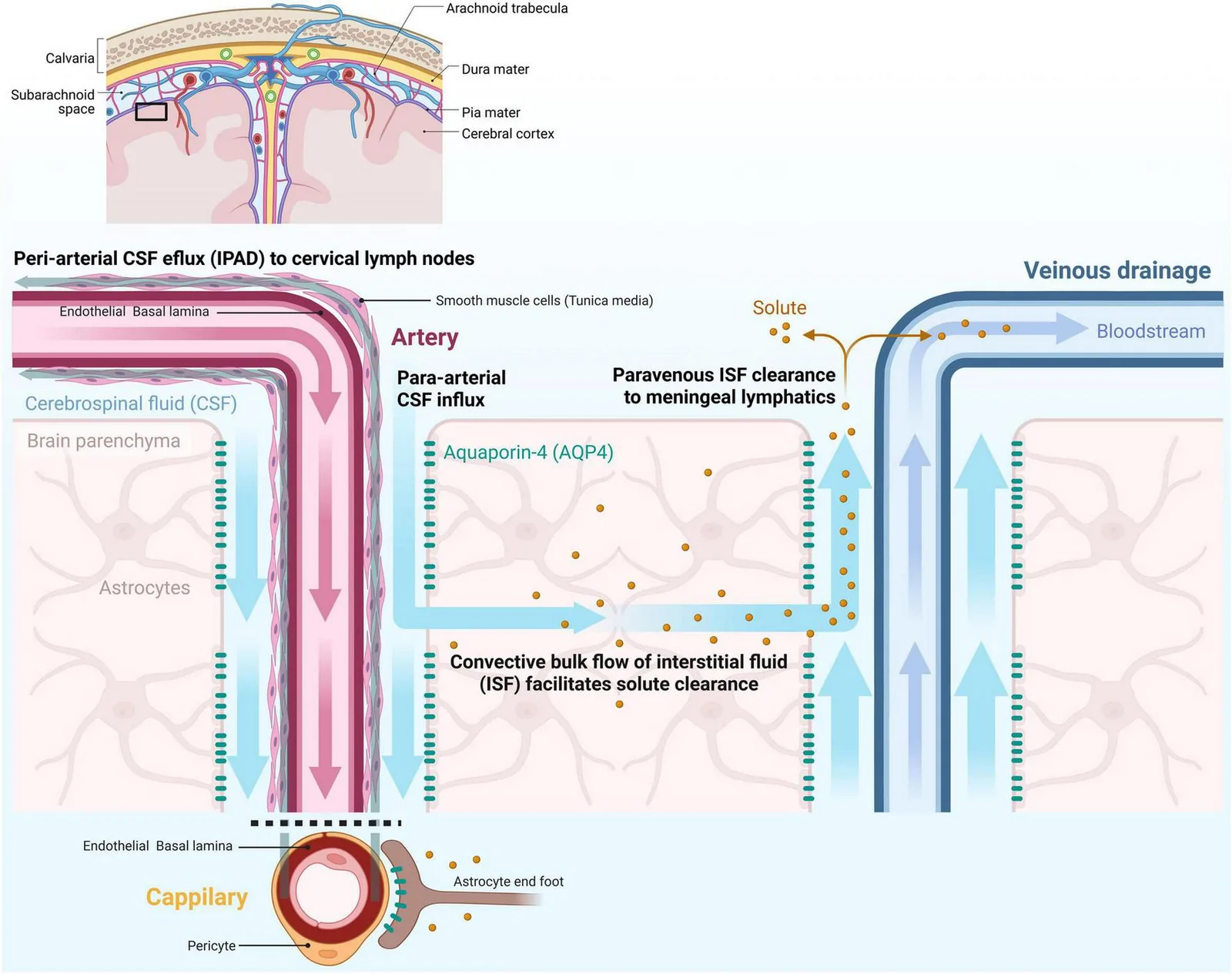
Peri- and paravascular drainage pathways. CSF enters the brain along the para-arterial space alongside the exterior wall of penetrating pial arteries, bidirectional fluid and solute exchange with the neuropil takes place through the astrocyte endfeets, mediated by AQP4, and then ISF flows out along the paravenous space. Moreover, a counter-current flow of ISF filtrates in the basement membranes of parenchymal capillaries, which continues through the thickness of the arterial tunica media basement membranes, until it diffuses around large arteries in the soft tissues of the head and neck and then is absorbed by blind-ended lymphatic capillaries embedded directly adjacent to these major vessels (artwork created in https://BioRender.com).

Para- and perivascular compartments of the CNS form a continuous vascular-associated network supporting CSF influx and efflux, directional molecular transport along vascular basement membranes, immune surveillance, while integrating interstitial fluid exchange with neuroimmune signaling processes (Hannocks et al., 2018; Ineichen et al., 2022; Ramaswamy et al., 2022) (Figure 3).

**FIGURE 4:**
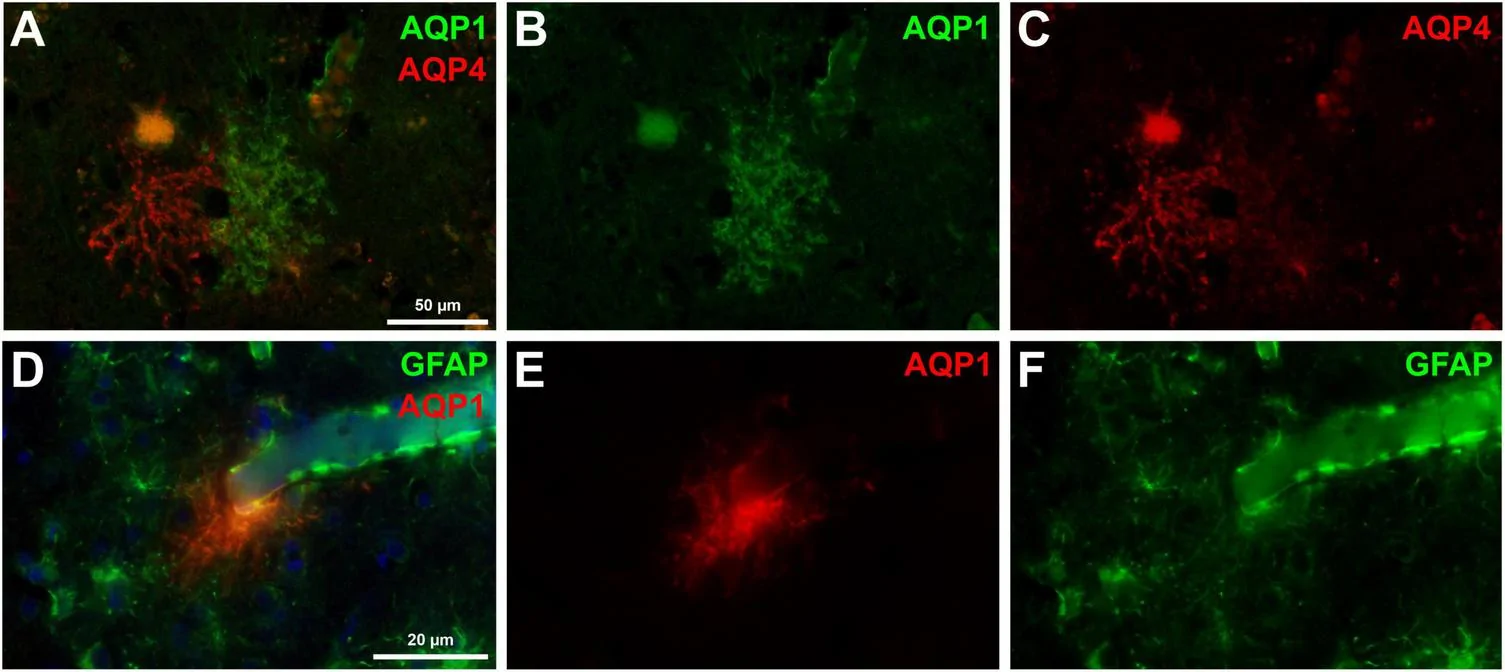
Aquaporins in the CNS. AQP1 and AQP4 are expressed in both parenchymal astrocytes **(A–C)**, as well as in perivascular astrocytes **(D–F)** in exemplary images from human aging patients; fluorescence double immunostaining – conventional fluorescence microscopy.

Anatomically, perforating subpial arteries enter the brain parenchyma initially enveloped by the pia mater. This path creates a paravascular compartment bounded internally by a pial connective tissue layer and externally by AQP4-rich astrocytic endfeet resting on a basement membrane (Weller et al., 2009; Jessen et al., 2015; Bacyinski et al., 2017). Moreover, within the structure of an artery, basement membranes do not exist only beneath the endothelium, but also wrap around smooth muscle cells of the tunica media, and between these smooth muscle cells and within this network of laminin and collagen fibers, a distinct fluid diffusing compartment has been identified, namely the perivascular space (Carare et al., 2008; Weller et al., 2009; Bakker et al., 2016; Morris et al., 2016). At the capillary level, the pial layer and muscle tunica media are no longer present, and the endothelium is instead surrounded by a fused vascular-glial basement membrane associated with astrocytic endfeet and pericytes (Morris et al., 2016; Voumvourakis et al., 2026). Thus, at this level, the perivascular and paravascular spaces join into the loose fibrillar meshwork of the capillary-glial basement membrane (Figure 3). As postcapillary venules are being formed, the fluid diffusing within the basement membranes drains into a paravenous space delineated between the venous tunica adventitia inside and the internal glia limitans on the outside. At the venous level, dedicated diffusion pathways within the tunica media are absent; nevertheless, the paravascular space is preserved in both arterial and venous domains, permitting continuous fluid and solute diffusion from regions of higher to lower pressure, according to fundamental diffusion principles. The paravascular arterial and venous spaces have been named collectively as the Virchow-Robin space, and are routinely evaluated in MRI brain imaging (Zhang et al., 1990; Cherian et al., 2016).

The above histological and anatomical basis led to an integrative view of fluid production and flow around and in the brain parenchyma. Thus, CSF is produced primarily by the choroid plexuses within the cerebral ventricles, where fenestrated capillaries and specialized epithelial cells regulate the secretion of fluid into the ventricular system. Although CSF production is classically described as a process of filtration and active secretion, the choroid plexus is now understood as a dynamic interface that contributes not only to CSF formation but also to the maintenance of CNS homeostasis through transport of ions, nutrients, signaling molecules, and waste products, with AQP1 as a central player (Jessen et al., 2015; Spector et al., 2015). From the lateral ventricles, CSF passes through the interventricular foramina into the third ventricle, then through the cerebral aqueduct into the fourth ventricle, before entering the SAS around the brain and spinal cord, through the foramina of Luschka and Magendie. Once in the SAS, CSF participates in a broader circulation that extends beyond the ventricular system. Thus, CSF enters the brain along paraarterial spaces, driven in part by arterial pulsatility, respiration, and sleep-related changes in interstitial dynamics (Iliff et al., 2012, 2013). The influx of CSF along these spaces facilitates convective exchange with ISF in the parenchyma, thereby supporting the clearance of soluble metabolites and proteins.

### 6.2 Glymphatic system

Within the brain parenchyma, CSF and ISF mix in a manner that is mediated by astrocytic endfeet AQP4 channels. This organization is thought to enhance fluid movement from paravascular spaces into the interstitial compartment and promote efficient solute transport through the neuropil (Iliff et al., 2012; Jessen et al., 2015). Waste products generated by neuronal and glial metabolism, including Aβ and tau-related species, are then mobilized from the interstitial space toward the paravenous efflux pathways, ultimately reaching dural sinuses, meningeal lymphatic vessels and extracranial lymphatic drainage routes (Aspelund et al., 2015; Louveau et al., 2015). This brain-wide route for CSF–ISF exchange has been deemed the glymphatic drainage pathway, and is believed to be more active during sleep, when interstitial space may expand and promote convective exchange, thereby enhancing metabolite removal (Xie et al., 2013). It interacts with other clearance mechanisms such as the trans-BBB efflux, intramural periarterial transport, and meningeal lymphatic drainage (Voumvourakis et al., 2026). Glymphatic drainage facilitates directional exchange between CSF and ISF, driven by arterial pulsatility, its convective clearance mechanism supplementing the diffusion-based transport (Jessen et al., 2015; Bacyinski et al., 2017; Rasmussen et al., 2018; Benveniste et al., 2019; Natale et al., 2021; Chen et al., 2025). Impairment of this system has been implicated in aging, TBI, and neurodegenerative disorders, particularly AD, where reduced clearance of Aβ contributes to protein accumulation and disease progression (Nedergaard and Goldman, 2020; Manescu et al., 2025).

AQP4 at astrocytic endfeet controls the exchange across the paravascular–interstitial interface rather than bulk propulsion. The data remain conflicting: some models show unchanged tracer kinetics after AQP4 deletion (diffusion-dominant behavior), while others demonstrate slower transfer from para-arteriolar spaces into the parenchyma without AQP4, suggesting a role in rapid transmembrane water movement (Wardlaw et al., 2020). This discrepancy stems most probably from methodological variations across animal models rather than mechanistic flaws, and indicate a need for more standardized protocols. Thus, the choice of anesthetic regimen modulates interstitial space and arterial pulsatility, as ketamine/xylazine preserve glymphatic influx, whereas volatile anesthetics like isoflurane suppress it, diminishing thus the impact of AQP4 deletion (Xie et al., 2013; Gakuba et al., 2018). Next, high-pressure or high-volume tracer injection create artificial hydrostatic gradients that overwrite subtle, AQP4-dependent flows (Iliff et al., 2012). Finally, structural adaptations in germline AQP4-knockout mice, such as altered baseline intracranial pressure, confound direct comparisons (Verkman et al., 2014). AQP4-dependent water flux regulates extracellular volumetric dynamics and solute redistribution independent of ion permeability, while loss of AQP4 polarization disrupts astrocyte–vascular fluid coupling, leading to expansion of the interstitial compartment and impaired perivascular washout with retention of activity-derived metabolites (Amiry-Moghaddam et al., 2004b; Cruz et al., 2013; Dmytrenko et al., 2013; Jin et al., 2013; Bakketun et al., 2026). Quantitative analyses further demonstrate that glymphatic efficiency is influenced by the molecular size of transported solutes, with perivascular AQP4 localization exerting a greater impact on clearance of large macromolecules compared with smaller diffusible compounds (Bojarskaite et al., 2024). Consistent with this, loss of endfoot complex integrity further reduces solute clearance, establishing AQP4 polarization, rather than expression level, as the principal determinant of convective clearance efficiency (Dai et al., 2017; Lopes et al., 2025). Reduction of AQP4 expression, loss of its perivascular polarization, age-related structural alterations, and sleep disruption converge as upstream determinants of glymphatic dysfunction (Silva et al., 2021). This results in diminished Aβ clearance, retention of neurotoxic aggregates within the interstitial space, directly contributing to AD pathogenesis (Jessen et al., 2015; Benveniste et al., 2019; Lee et al., 2020; Natale et al., 2021; Thipani Madhu et al., 2024; Chen et al., 2025).

Considering that during the day-night cycle interstitial space can vary in volume with up to 60%, sleep emerges as a critical enhancer of glymphatic performance, promoting interstitial expansion followed by a coordinated amplified ISF outflow, including Aβ and tau-related species (Xie et al., 2013; Yang et al., 2013; Natale et al., 2021; Rehman et al., 2024). Sleep–wake cycling strongly regulates AQP4-dependent fluid dynamics, which declines with aging, as reduced sleep-dependent activity diminishes ISF movement and downstream drainage (Jessen et al., 2015). Chronic sleep fragmentation, a pattern frequently associated with aging and AD, reduces slow-wave sleep continuity and impairs glymphatic clearance through several convergent mechanisms, including altered AQP4 expression, disturbed astrovascular coupling, reactive astrogliosis, and remodeling of perivascular spaces (Deng et al., 2024). In 5xFAD mice [expressing the Swedish (K670N/M671L), Florida (I716V), and London (V717I) mutations in APP, and the M146L and L286V mutations in PSEN1], sleep fragmentation has been associated with increased cognitive deficit, Aβ accumulation and altered patterns of AQP4 expression, further supporting the concept that disrupted sleep may accelerate AD pathology by weakening the AQP4-dependent glymphatic activity (Vasciaveo et al., 2023).

AQP4 also loses its astrocyte end feet expression with increasing age and dementia, and the degree of AQP4 depolarization correlates with amyloid burden, neurofibrillary pathology, and cognitive decline prior to dementia onset, suggesting that loss of astroglial polarity is an early pathological feature rather than a late phenomenon (Simon et al., 2022). Unlike AQP4, which predominantly governs parenchymal water redistribution and glymphatic clearance, AQP1 exerts upstream control at fluid-secreting interfaces, regulating CSF formation. This distinction reiterates CNS fluid regulation as a production versus clearance paradigm, rather than as a homogeneous water buffering system.

### 6.3 Intramural periarterial drainage pathways

In parallel with the glymphatic model, an important and related concept is intramural periarterial drainage (IPAD), which refers to the drainage of ISF and soluble waste products, along the periarterial pathway, in an opposite direction to the arterial blood flow, from pre-capillary arterioles to small arteries, and toward larger leptomeningeal arteries and then out of the brain itself (Weller et al., 2008; Morris et al., 2016) (Figure 3). Fluid movement within perivascular pathways occurs in a retrograde direction relative to blood flow, with ISF and solutes being cleared from the brain parenchyma toward larger arteries. This process involves the transport of ISF and solutes, including Aβ, along the basement membrane feltwork rather than through open fluid-filled spaces (Hannocks et al., 2018). While early theories proposed arterial pulsations from cardiac activity as the driving force for fluid movement along cerebral vessel basement membranes, these were found to be insufficient for generating adequate flow; instead, vasomotion- the cyclic contraction and relaxation of cerebrovascular smooth muscle cells- has been established as the primary driving mechanism of IPAD, with activity-dependent arteriolar dilation further supporting intramural transport along vascular basement membranes (Diem et al., 2018). The efficiency of IPAD is highly sensitive to the structural and mechanical properties of the arterial wall (Diem et al., 2017; Sun et al., 2022). Molecular organization of these vascular basement membranes is characterized by distinct laminin isoforms and structural proteins, reflecting arterial specialization and enabling IPAD (Hannocks et al., 2018). In this context, the basement membrane behaves as a poroelastic medium, and asymmetric permeability within the drainage pathway is crucial for effective retrograde transport (Diem et al., 2017; Aldea et al., 2019). Because these compartments are microscopic and difficult to access directly with conventional neuroanatomical techniques, computational approaches have been used to model solute movement within the basement membrane network. Such models suggest that IPAD-mediated transport along vascular basement membranes may proceed more efficiently than passive diffusion through the extracellular space, reinforcing the concept that IPAD represents an active and anatomically specialized clearance route for Aβ rather than a nonspecific perivascular diffusion process (Diem et al., 2016). Age-related stiffening, arteriosclerosis, or vascular injury compromise basement membrane integrity and reduce vasomotion amplitude, leading to impaired clearance of interstitial solutes (Diem et al., 2017; Sun et al., 2022). Experimental disruption of IPAD results in dilatation of perivascular spaces, reduced ISF clearance rates, and accumulation of extracellular matrix alterations that further impede intramural flow (Sun et al., 2022). Pericyte loss induces BBB breakdown, with direct leakage from capillary–arteriolar segments into the perivascular compartments, leading to their expansion (predominantly in white matter), and functionally overloading of the periarteriolar drainage pathway. In AD, similar permeability defects support a mechanism in which vascular extravasation exceeds IPAD-mediated clearance capacity, resulting in fluid retention and impaired solute outflow (Wardlaw et al., 2020).

Although both the glymphatic system and the intramural periarterial drainage contribute to Aβ removal, they represent distinct anatomical and functional clearance routes relying on the integrity of cerebral vasculature, and influenced by aging, sleep, arterial pulsatility, and astroglial polarization and integrity. While the glymphatic pathway is primarily a CSF-driven exchange system which terminates with the paravenous outflow of CSF-ISF, IPAD refers to the removal of ISF and soluble wastes along the arterial basement membranes, in an opposite direction to the arterial blood flow. As a result, failure of glymphatic clearance promotes retention of soluble Aβ within the interstitial compartment as plaques, and in the perivascular glia limitans as double barreling and dysphoric angiopathy-type of accumulations, whereas impaired IPAD favors deposition of Aβ within vascular basement membranes as CAA (Figure 1) (Tarasoff-Conway et al., 2015). Initial Aβ deposition patterns concur with this theory, localizing preferentially within periarteriolar and capillary walls while sparing venules, and further suggesting the functional relevance of arterial-associated pathways (Kumar-Singh et al., 2005; Weller et al., 2008; Morris et al., 2016; Wardlaw et al., 2020). Furthermore, failure of both the glymphatic pathway and the IPAD system leads to complex patterns of parenchymal and vascular/perivascular Aβ accumulations, depending on the sporadic determinants of AD, or the mutational background leading to variate vascular affinity patterns of the Aβ peptide (De Jonghe et al., 1998; Aldea et al., 2019; Bonnar et al., 2025).

### 6.4 Venous drainage

**FIGURE 5:**
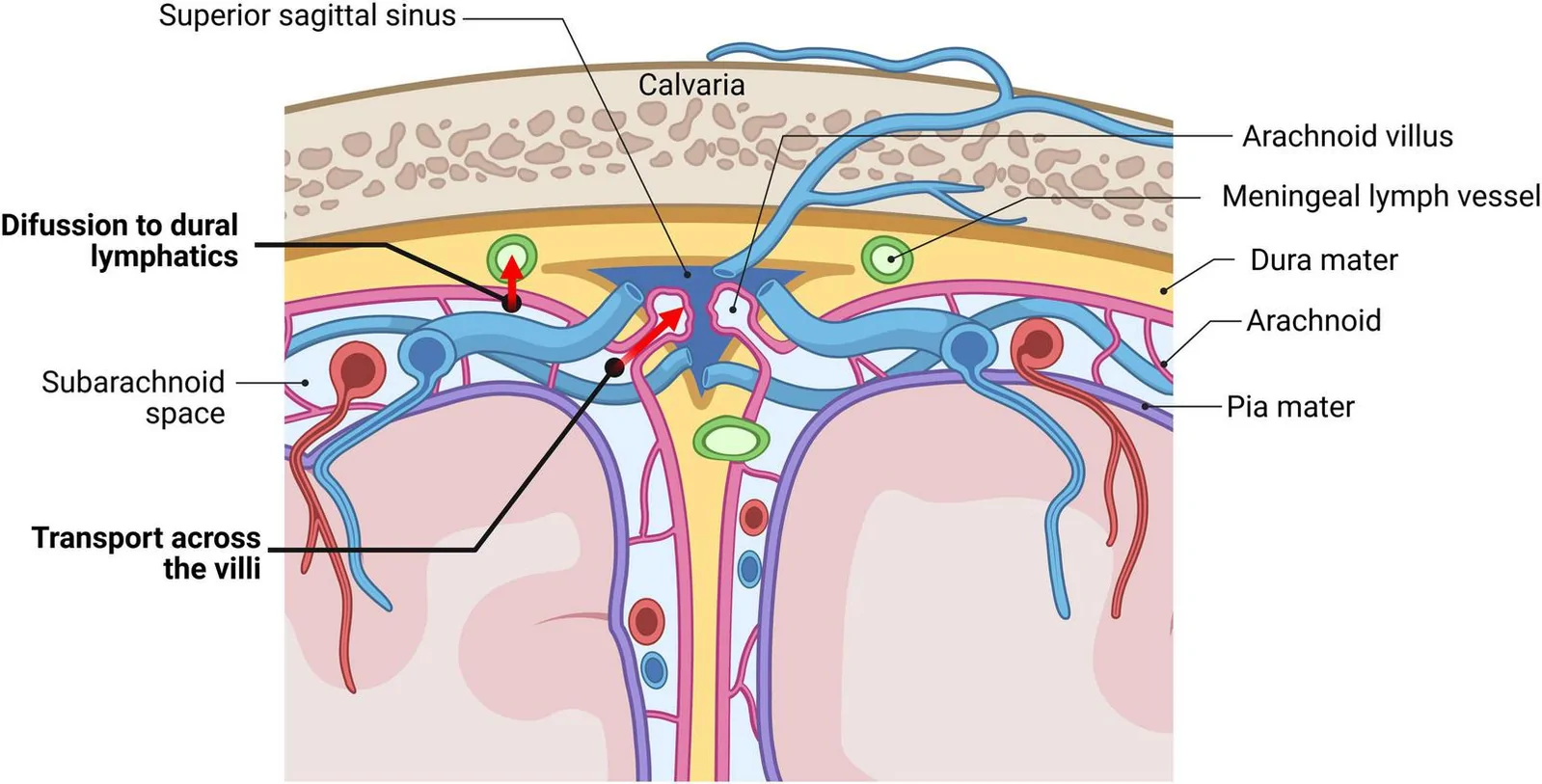
Meningeal venous and lymphatic CSF drainage pathways. CSF from the subarachnoid space drains into the dural sinuses via the arachnoid villi, but can also diffuse toward the intradural/ subdural lymphatic vessels, ultimately draining toward the venous system and respectively the lymph nodes at the base of the skull (artwork created in https://BioRender.com).

Histologically, arachnoid villi (Pacchioni granulations) are composed of arachnoid cells organized into trabecular and villous structures covered by a cellular layer continuous with the arachnoid membrane. These structures traverse the dura and project into venous sinuses without a direct mixing of CSF and blood under normal conditions (Pollay, 2010; Proulx, 2021) (Figure 5). Their interface with the sinus wall is highly specialized: the venous endothelium and surrounding connective tissue provide a semipermeable barrier that supports selective fluid transfer while limiting the passage of formed blood elements. Electron microscopic studies have shown vacuolated cells and transcellular channels within the granulations, supporting the idea that CSF movement may occur through a combination of paracellular, transcellular, and vesicular transport mechanisms rather than through a single open conduit (Tripathi and Tripathi, 1974; Pollay, 2010).

Functionally, arachnoid villi act as pressure-sensitive CSF drainage sites. CSF flows from the SAS into the venous circulation when CSF pressure exceeds venous sinus pressure, establishing a one-way, valve-like mechanism, and this pressure gradient is critical for maintaining CSF volume and intracranial pressure within physiological limits (Pollay, 2010; Proulx, 2021). When venous pressure rises or villous outflow becomes impaired, CSF absorption may decrease, contributing to raised intracranial pressure. Conversely, the efficiency of villous drainage helps protect against CSF accumulation and supports steady-state fluid homeostasis in the cranial compartment (Sakka et al., 2011; Proulx, 2021). From a mechanistic perspective, arachnoid villi are not simply passive routes in the meninges. Rather, they are dynamic structures whose transport properties depend on microanatomical architecture, pressure gradients, and the permeability characteristics of the arachnoid-dural-venous interface (Pollay, 2010; Tripathi and Tripathi, 1974). Some models suggest that CSF first enters endothelial vacuoles within the villous cells before being transferred into the venous sinus lumen, while other data support passage through intercellular clefts under favorable pressure conditions (Tripathi and Tripathi, 1974; Pollay, 2010). Moreover, AQP1 has also been showed to be expressed into arachnoid cells and it may contribute to the expansion of arachnoid cysts by facilitating the movement of fluid into the cyst lumen (Kurokawa et al., 2023).

Anatomically, although small arachnoid villous structures may appear earlier in life, larger macroscopic granulations typically become more prominent with age, reflecting maturation of the CSF outflow system through the adult life (Pollay, 2010; Sakka et al., 2011). Clinically, dysfunction or obstruction of arachnoid villous drainage has been implicated in disorders of CSF circulation, including communicating hydrocephalus and impaired intracranial pressure regulation (Sakka et al., 2011; Proulx, 2021). On the other hand, there is also clear evidence suggesting that alternative CSF draining routes are fully functional, and in this direction, a MRI-based study on 120 subjects with ages varying between neonate to 80 years of age, indicates that arachnoid granulations are largely absent at birth, peak around 40 years of age, and do not appear to be essential for CSF absorption, as many individuals with normal intracranial pressure lack these structures in the dural sinuses (Rados et al., 2021). In the same line is also the fact that arachnoid granulations may calcify with age, as these are well recognized on CT as incidental findings (Roche and Warner, 1996). These suggest that arachnoid villi undergo remodeling over time rather than simply functioning as stable, dominant drainage valves throughout life, and points out to the growing recognition of alternative CSF outflow pathways [2,4]. Because these pathways can transport CSF and solutes to the cervical lymph nodes, many authors now argue that CSF clearance is distributed across multiple routes rather than being dominated by arachnoid villi alone [2,4]. In this broader framework, arachnoid villi may represent one important route among several, with relative contribution varying by species, age, physiological state, and disease.

### 6.5 Dural lymphatic drainage

The classical anatomical view describing that most of the CSF clearance occurs by venous drainage through arachnoid villi projecting into the dural venous sinuses, has been expanded in the recent years, as physiological and imaging evidence show that tracers injected into the CSF can also be found within lymphatic vessels draining from the cranium and spine, and surrounding the cranial nerves (Proulx, 2021).

The discovery of lymphatic vessels within the dura mater of mice, humans and monkeys resolved a long-standing question about how brain-derived fluid and immune cells reach peripheral lymph nodes (Aspelund et al., 2015; Absinta et al., 2017). Unlike the glymphatic system, which describes fluid movement within the brain and along paravascular routes, meningeal lymphatics provide a direct lymphatic outflow pathway from the cranial compartment, and are now seen as part of a broader brain-wide clearance network, essential for both waste clearance and immune surveillance (Chachaj et al., 2023; Zhang et al., 2025; Zhao et al., 2025). Although still under debate, these vessels seem to be located primarily within the dura mater along the dural venous sinuses and skull base, where they collect CSF-associated material and immune cells before conveying them to the deep cervical lymph nodes (Aspelund et al., 2015; Louveau et al., 2015; Raper et al., 2016) (Figure 5).

Basal meningeal lymphatics are especially important for clearing large molecules, and age-related decline in their structure and function provides a mechanistic link between aging, impaired clearance, and neurodegeneration (Ahn et al., 2019). Aβ reaches meningeal lymphatic vessels through pathways interfacing with the BBB and perivascular compartments (Dupont et al., 2020). Functionally, they form a dural network receiving glymphatic-derived fluid flux, coupling perivascular CSF–ISF exchange to extracranial lymphatic drainage and thereby integrating CNS clearance pathways (Kim and Kipnis, 2025). In parallel, they enable antigen transport and controlled immune cell trafficking, including T lymphocyte–mediated surveillance without undergoing inflammatory expansion (Louveau et al., 2018). Age-related deterioration induces functional impairment of meningeal lymphatic drainage and reduces vessel transport efficiency, disrupting this clearance–immune coupling through increased interferon-γ signaling (Da Mesquita et al., 2018; Feng et al., 2025). Reduced lymphatic transport capacity limits this efflux, promoting the retention and aggregation of Aβ within brain compartments, while targeting interferon-γ–dependent mechanisms may restore drainage efficiency (Dupont et al., 2020; Chachaj et al., 2023; Rustenhoven et al., 2023; Feng et al., 2025). In AD, disruption of this lymphatic drainage–immune interface impairs both fluid clearance and immune homeostasis, a process exacerbated by age-related structural and molecular deterioration of meningeal lymphatic vessels, which reduces vessel integrity and drainage efficiency, impairing the clearance of CSF–borne metabolites, and promoting retention of solutes within upstream interstitial and perivascular compartments (Zhao et al., 2025; Elyse Moore et al., 2026). This leads to consequent accumulation of Aβ and tau in both meningeal compartments and brain parenchyma, accompanied by enhanced neuroinflammatory signaling and pro-inflammatory cytokine production, affecting glial and neuronal activity, thereby driving disease progression (Chachaj et al., 2023; Guo et al., 2023; Rego et al., 2023).

Proof-of-principle studies have underlined the importance of meningeal lymphatics and of the glymphatic-lymphatic functional cooperation in maintaining physiological levels of ISF outflow. Thus, ablation of meningeal lymphatics in mice, followed by intracisternal injection of fluorescently labeled ovalbumin, lead to significantly reduced tracer drainage from the CSF to deep cervical lymph nodes (Da Mesquita et al., 2018). Moreover, surgical ligation of deep cervical lymph nodes on AQP4 knockout mice arrested both glymphatic and meningeal lymphatic drainage, providing a definitive proof of their synchronous functionality (Cao et al., 2018). Furthermore, in 5xFAD mice, increased Aβ vascular deposition is accompanied by a progressive dysfunction of meningeal lymphatic vessels, and ablation of meningeal lymphatics in this background accelerated Aβ deposition and accentuated the cognitive and memory impartments (Da Mesquita et al., 2021). Treatment of these animals with vascular endothelial growth factor C (VEGF-C) restored lymphatic function and improved Aβ clearance, underlying the strong continuity of these two systems.

In summary, the meningeal lymphatic network serves as the terminal clearance pathway for ISF and its soluble fractions, and a loss in either downstream lymphatic transport capacity itself or upstream AQP4-mediated glymphatic clearance, lead to a global loss-of-function of the meninges-associated lymphatic system.

### 6.6 Peri-neural drainage

**FIGURE 6:**
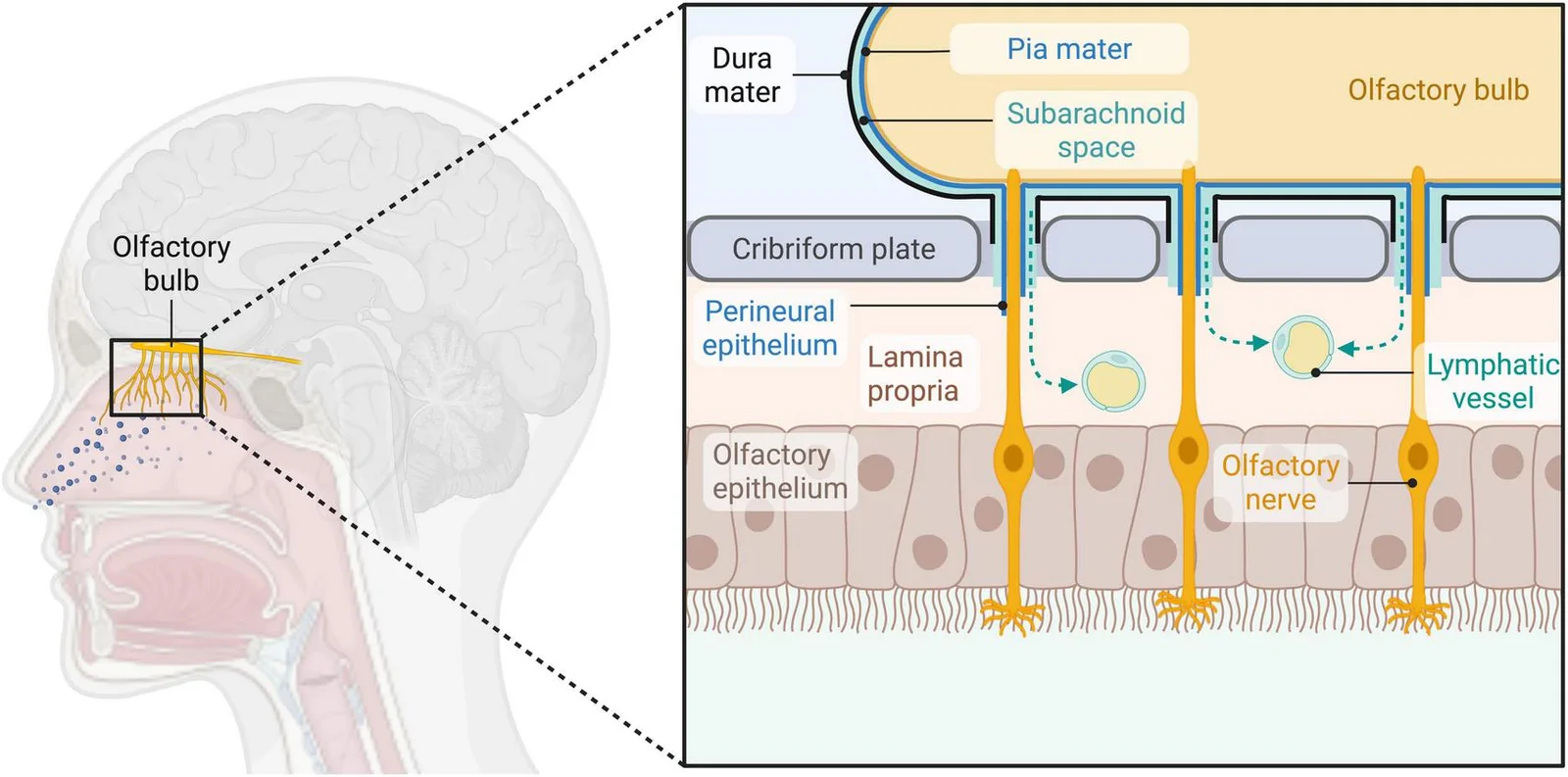
Olfactive perineural drainage pathway. Solute-laden CSF drains around the subarachnoid space of the olfactory bulb, then around the olfactory nerves which descend through the cribriform plate together with a meningeal sheeting on their initial portion, diffuses in the submucosa and lamina propria of the olfactory mucosa, and finally is collected through the nasal lymphatics (green dotted arrows). Artwork created in https://BioRender.com.

In parallel, another important outflow route has been described along cranial nerves, and especially around the olfactory nerves (Faber, 1937; Walter et al., 2006; Proulx, 2021). This perineural drainage pathway along the olfactory nerve meningeal sheet is considered a primary site for CSF outflow from the SAS toward the extracranial lymphatic system, and involves CSF moving along olfactory nerve sheaths, through the cribriform plate of the ethmoid bone, to reach lymphatic vessels of the nasal submucosa through the loose connective tissue lamina propria (Proulx, 2021) (Figure 6).

More in-depth analysis even described several potential models for how CSF transitions from the perineural space to the submucosal lymphatics. Proposed mechanisms include: (i) the existence of discontinuous perineural sheaths around the olfactory nerve fibers (fila olfactoria) once they pass through the cribriform plate, with CSF diffusing into the loose connective tissue of the nasal submucosa, where it is subsequently absorbed by forming lymphatic vessels (Walter et al., 2006); (ii) a more direct route where lymphatic vessels form a “collar” or fuse with the perineural sheath, allowing CSF to enter directly the lymphatic lumen without significant exposure to the interstitial space (Johnston et al., 2004), and (iii) a trans-cribriform lymphatics pathway, with lymphatic vessels that actually cross the cribriform plate alongside nerve bundles to directly access the CSF at locations where meningeal barriers are discontinuous (Hsu et al., 2019). In the case of a direct route between the lymphatic vessels and the perineural space, this would not be the only place in the organisms with such an interface, as the peritoneal cavity mesothelial cells contains stomata and express AQP1 in order to allow for a direct drainage of the peritoneal fluid toward adjacent lymphatic vessels (Lai et al., 2001), but for the moment the molecular mechanisms of this direct route are still under scrutiny. The relative importance of the nasal route in humans remains debated, because *in vivo* human imaging has not always shown substantial tracer enrichment in the nasal mucosa despite strong signal near the cribriform plate (Mehta et al., 2021), or as there have been suspicions that the pressure used in injecting tracers within the cranial nerve peripheral sheets might have caused artefactual ruptures and false communications (Faber, 1937).

Altogether, growing evidence supports the existence of an integrated glymphatic–venous–lymphatic continuum in which arterial pulsatility, venous outflow, and meningeal lymphatic drainage operate as a unified clearance network. Disruption of any component – loss of AQP4 polarization, venous insufficiency, or lymphatic dysfunction – can propagate system-wide failure, leading to protein accumulation, sterile inflammation, fluid retention, and increased vulnerability to vascular injury (Costea et al., 2025).

## 7 AQP4 modulation in animal models of AD

### 7.1 Pharmacological AQP4 modulation in AD animal models

AQP4 has come to represent a key therapeutic target in CNS disorders linked to impaired ISF drainage, with pharmacological approaches either limiting AQP4-dependent water flux or restoring its perivascular organization and clearance-related functions. Both AQP4 expression levels and its predominant perivascular localization are essential for maintaining BBB integrity and glymphatic function, while histopathological studies have demonstrated loss of perivascular AQP4 polarization in both human AD and experimental AD mouse models (Simon et al., 2022). As a consequence, techniques that aim to restore the expression levels and polarization of AQP4 have been sought in order to improve the impaired perivascular transport and enhance Aβ drainage in different models of AD (Mohaupt et al., 2023).

Glucagon-like peptide-1 receptor (GLP-1R) signaling represents an indirect regulatory pathway influencing AQP4-dependent clearance mechanisms in AD. This effect is mediated through modulation of AQP4 subcellular distribution rather than changes in total expression. Liraglutide induces protein kinase A (PKA)-dependent phosphorylation of AQP4, driving its relocalization to perivascular astrocytic endfeet in reactive astrocytes (Sasaki et al., 2024). 1-month daily administration of the GLP-1R agonist liraglutide in an APP NL-G-F Knock-in transgenic mouse model of AD, that expresses humanized mutant APP at physiological levels, showed a clear reduction of Aβ42 deposits in the cerebral cortex of these animals, and improved their spatial memory tests. In this setup, AQP4 localization in reactive astrocytes extended from perivascular endfeet to perivascular astrocyte cell surface, a subcellular polarization phenomenon that might explain a better clearance of the soluble Aβ across the perivascular/paravascular drainage pathways (Sasaki et al., 2024).

**FIGURE 7:**
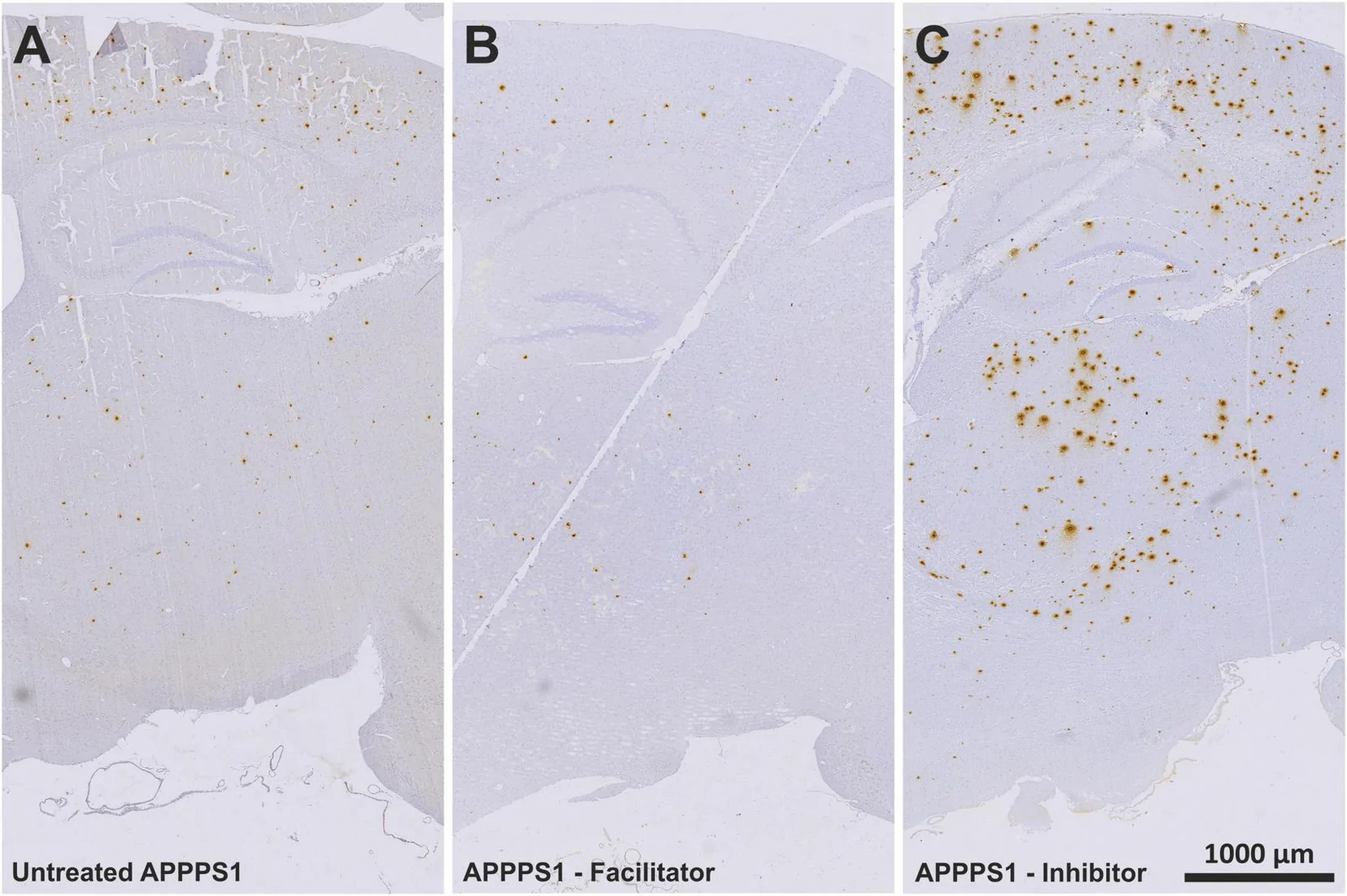
AQP4 modulation controls plaque burden in an APPPS1 mouse model **(A)**. The treatment with the TGN-073 AQP4 facilitator decreases total plaque load **(B)**, while pharmacological inhibition with the TGN-020 AQP4 inhibitor increases amyloid accumulation **(C)**; immunohistochemistry for total Aβ.

A single dose of the AQP4 inhibitor TGN-020 [N-(1,3,4-thiadiazol-2-yl)-3-pyridinecarboxamide] reduces astrocyte-dependent Aβ clearance around the blood vessels and increases perivascular accumulation of intraparenchymally injected soluble Aβ40 which become trapped in its perivascular clearing route, and demonstrated its direct impairing effect on peri- and paravascular elimination pathways (Rosu et al., 2020). One month daily intraperitoneal administration of the TGN-020 inhibitor on amyloid precursor protein/presenilin-1 double transgenic mice (APPPS1) mice further confirmed this dependency, by increasing the amyloid burden and decreasing memory performances compared to untreated APPPS1 mice (Zhou et al., 2023; Manescu et al., 2025) (Figure 7). Moreover, a daily treatment for 1 month with the AQP4 facilitator TGN-073 [N-(3-(Benzyloxy)pyridin-2-yl) benzene-sulfonamide] in an APPPS1 transgenic mouse model resulted in a significant reduction of total Aβ burden, encompassing both fibrillar and soluble species, together with a decrease in the Aβ40/Aβ42 ratio and improved behavioral performance, supporting the clearance-enhancing effect of AQP4 amplified function (Manescu et al., 2025). In the same experimental setup, the pharmacological inhibition of AQP4 on the same APPPS1 background disrupted the CSF–ISF exchange and reduced clearance efficiency, promoting progressive accumulation of amyloid plaques (Manescu et al., 2025). This head-to-head comparison of both the facilitator and the inhibitor, on the same genetic background represent in fact a proof-of-concept preclinical evidence for AQP4 facilitation as a disease-modifying approach in amyloid pathology. Although TGN-020 and TGN-073 have been confirmed to modulate ISF fluid drainage according to the postulated mechanistic pathways, including experiments on animal models of CNS oedema and transgenic AD models, both compounds need further confirmation of their specificity for future human applications. More specifically, TGN-020 has been suggested not to be in fact specific for the AQP4 pore itself, but rather to act on its upstream signaling pathway, and this raises the possibility that some observed effects reflect altered membrane properties, cellular signaling, or assay-specific interference rather than selective pore blockade (Unger et al., 2024). In fact, as illustrated above, AQP4 functions extend beyond water transport, including astrocyte endfoot organization, ion homeostasis, neuroinflammatory signaling, and interactions with dystrophin-associated complexes. Moreover, for both compounds, minimal effective dosages for animal model studies or concentration-related nonspecific effects have not been studied. It is thus conceivable that resulting phenotypes might in fact also include unaccounted for effects in membrane integrity, cell volume regulation, vascular tone, inflammatory signaling, or mitochondrial function.

To complicate things even further, a special type of transcription past the stop codon, a programmed readthrough has been identified in the transcript of AQP4, generating a C-terminally elongated variant of AQP4 (AQP4X), an isoform which has been localized exclusively perivascular, compared to the normal-length AQP4 which is also expressed in the astrocyte peri-nuclear membrane, and not only in the vicinity of blood vessels (De Bellis et al., 2017; Sapkota et al., 2019). In this line of evidence, two candidate AQP4X enhancers, apigenin and sulphaquinoxaline, injected into the hippocampus of an APPPS1 mouse model showed a significantly enhanced elimination of Aβ into the ISF compared to untreated transgenic mice, proving that AQP4X facilitation enhances perivascular fluid flow (Sapkota et al., 2022). Moreover, although human AD casuistry and mouse model studies show an increase in total AQP4 expression levels, this is in fact accompanied by a decreased AQP4X/AQP4 ratio, underlying the essential loss of perivascular drainage function linked with the downregulation of AQP4X (Zeppenfeld et al., 2017; Sapkota et al., 2022).

These findings highlight the complexity of AQP4 pharmacological modulation, which depends on compound-specific mechanisms ranging from direct channel inhibition to indirect regulation via intracellular signaling pathways, underscoring their relevance in AD.

### 7.2 AQP4 overexpression in AD animal models

The effects of AQP4 overexpression are highly dependent on its spatial organization, as diffuse upregulation may promote CNS edema through enhanced vascular permeability, whereas increased expression associated with preserved perivascular polarization enhances ISF clearance and supports BBB integrity.

Transgenic mice with glial-specific AQP4 overexpression (∼2.3-fold increase driven by an astrocyte promoter) display normal baseline neuroanatomy and behavior, but under osmotic stress these animals develop markedly accelerated cytotoxic edema in a dose-dependent manner, directly linking increased but depolarized astroglial AQP4 levels to brain water accumulation and intracranial pressure elevation (Henn and Hamberger, 1971). In this context, driving AQP4 overexpression using an adeno-associated virus approach with an astrocyte-specific promoter in APPPS1 Tg2576 mice leads to its distribution toward non-perivascular fine astroglial processes instead of the perivascular endfeet. Importantly, this pattern of overexpression neither improved perivascular clearance nor reduced parenchymal Aβ accumulation (Simon et al., 2022). Collectively, these results indicate that preserved perivascular AQP4 polarization is more critical for effective perivascular ISF and Aβ clearance than increased expression in non-perivascular astrocytic domains.

### 7.3 AQP4 knockout in AD animal models

In APPPS1 mice, knocking out AQP4 disrupts astrocytic endfeet–dependent fluid exchange and impairs Aβ clearance, resulting in increased parenchymal accumulation and CAA, indicating failure of both interstitial and periarterial elimination pathways (Xu et al., 2015). In 12-month-old animals, loss of AQP4 is associated with worsened cognitive performance and increased parenchymal Aβ deposition, together with reduced synaptic protein levels and brain-derived neurotrophic factor in the hippocampus and cortex (Xu et al., 2015). This is further linked to astrocytic atrophy and reduced interleukin-1 beta (IL-1β), interleukin-6 (IL-6) and tumor necrosis factor-alpha (TNF-α), demonstrating impaired astroglial activation and loss of astrocyte-mediated support for Aβ removal (Xu et al., 2015).

In 5xFAD mice, genetic deletion of AQP4 leads to increased cortical amyloid deposition, with a shift toward a higher proportion of fibrillar plaques relative to dense-core structures (Pedersen et al., 2023). AQP4 deficiency is further associated with reduced density of glial fibrillary acidic protein (GFAP)-positive peri-plaque astrocytic processes and decreased microglial coverage of plaques, indicating disruption of the coordinated glial response. Disruption of AQP4, either through gene deletion or loss of its polarized perivascular localization, hinders Aβ removal and facilitates plaque formation (Pedersen et al., 2023). These structural alterations are accompanied by enhanced neuronal uptake of Aβ and increased number of dystrophic neurites in the neuropil surrounding the plaques (Smith et al., 2019). Moreover, AQP4 gene deletion in the 5xFAD background induces a progressive, age-dependent decline in behavioral performance, characterized by a reduction in nocturnal locomotor activity between 20 and 36 weeks. This phenotype is accompanied by motor impairment and increased neuronal excitability, reflected by spontaneous convulsive episodes across all AQP4-deficient animals, occurring without detectable changes in parenchymal Aβ deposition, peri-plaque astrocytic reactivity, or microglial activation relative to transgenic controls. This dissociation between amyloid burden and neuronal phenotype in AQP4-deficient 5xFAD mice is expressed along a defined temporal trajectory of behavioral decline (Padua et al., 2024), and its loss unmasks a distinct disease phase characterized by neuronal hyperexcitability downstream or independent of plaque accumulation (Abe et al., 2020; Barbour et al., 2024).

AQP4 anchoring at astrocytic endfeet is mediated by postsynaptic density protein 95/disc large tumor suppressor/zonula occludens-1 (PDZ)-dependent interaction with α-syntrophin within the dystrophin-associated complex, underlying one more time that AQP4 spatial organization, rather than absolute protein abundance alone, governs glymphatic transport (Hoddevik et al., 2017; Pedersen et al., 2023). In α-syntrophin knockout (Snta1-/-) mouse model, total AQP4 expression is preserved but redistributed away from the endfeets, producing a depolarization phenotype with significant loss of perivascular AQP4 (∼79–94%), and consequent disruption of the clearance-relevant compartment (Hoddevik et al., 2017). Loss of perivascular AQP4 polarization in AQP4-/- and snta1-/- models reduces CSF influx and interstitial solute transport (Hoddevik et al., 2017; Mestre et al., 2018), and α-syntrophin deficiency produces increased Aβ accumulation and exacerbation of plaque pathology (Simon et al., 2022). Functionally, α-syntrophin–dependent polarization rather than total AQP4 expression determines convective interstitial transport, as mislocalization disrupts vectorial perivascular water flux and produces a more pronounced augmentation of amyloid deposition compared to total AQP4 deletion (Pavlin et al., 2017; Pedersen et al., 2023). Thus, compared to the 5xFAD x AQP4−/− model, crossing 5xFAD mice with the Snta1−/− line results in a more severe accumulation of parenchymal and microvascular Aβ deposits, as well as amplified cognitive decline, proving that perivascular anchoring of AQP4 appears more critical for Aβ clearance than total channel expression alone (Pedersen et al., 2023). The extent of this loss-of-function and the fact that the AQP4 mislocalization phenotype cannot cope even with milder Aβ production is underlined by the crossing of the less severe APPPS1 Tg2576 model with the *Snta1*−/− background, which slowed down CSF tracer influx and ISF tracer efflux with increased Aβ accumulations compared to the APPPS1 model alone (Simon et al., 2022). Considering the exclusive perivascular localization of the AQP4X isoform, moderately increasing the AQP4X-to-total-AQP4 ratio enhanced the perivascular localization of both AQP4 and α-syntrophin without changing absolute protein expression, but increasing this ratio to 100% disrupted polarization and formation of higher-order complexes (Mueller et al., 2023). This demonstrates that a precise stoichiometric balance of isoforms is required to preserve AQP4’s structural integrity and functional properties.

The effect of AQP4 deletion extends beyond Aβ clearance to all diffusing solutes normally being drained away around the vasculature, thus in the rTg4510 tauopathy transgenic model, loss of perivascular AQP4 polarization at astrocytic endfeet impairs CSF–ISF exchange and glymphatic inflow–outflow dynamics, resulting in reduced tau clearance and increased extracellular tau retention (Harrison et al., 2020). These effects are further exacerbated by pharmacological AQP4 inhibition (Harrison et al., 2020), directly linking AQP4-dependent clearance failure with a time-dependent coupling between tau burden and functional decline (Wang et al., 2018). In early disease stages, phosphorylated tau accumulates in the absence of behavioral deficits, indicating that AQP4-independent clearance mechanisms are initially sufficient to buffer tau load before the onset of overt dysfunction (Xolalpa-Cueva et al., 2022). As AQP4-dependent clearance becomes insufficient, tau accumulation is associated with synaptic compartmentalization, dendritic spine loss, and reduced synaptic protein expression, defining a neuronal vulnerability phenotype that reflects impaired glymphatic-mediated protein removal (Kopeikina et al., 2013). Consequently, these alterations are associated with locomotor hyperactivity, spatial memory impairment and deficits in associative learning (Kishimoto et al., 2025) that tightly correlate with neurofibrillary tangle load, hyperphosphorylated tau levels, and brain atrophy (Blackmore et al., 2017; Wang et al., 2018).

## 8 AQP1 modulation in relationship to Aβ metabolism and animal models of AD

### 8.1 Pharmacological AQP1 modulation and Aβ metabolism

Pharmacological modulation of AQP1 has been showed to target both its control over osmotic water permeability at the levels of choroid plexuses and blood vessels, as well as its ion channel function, thereby modulating CSF production and flow, with downstream effects on perivascular clearance pathways. However, due to the fact that AQP4 is responsible for most of the water buffering around the brain blood vessels, AQP1 pharmacological modulation has not been directly targeted in the context of Aβ or tau overproduction, and therefore only mechanistic-driven hypotheses can be envisaged at this time.

Acetazolamide represents a sulfonamide compound that directly inhibits AQP1 by reducing osmotic water permeability, as evidenced by decreased cell swelling under hypoosmotic conditions, consistent with direct channel-level suppression of water flux (Gao et al., 2006). At the level of CSF dynamics, acetazolamide induces a rapid but transient reduction in AQP1 expression in choroid plexus epithelial cells (Pattisapu et al., 2010) and decreases trans-epithelial fluid flow (Pattisapu et al., 2010; Ameli et al., 2012), indicating that acetazolamide-mediated AQP1 inhibition directly alters CSF production, and altogether overall brain parenchyma outflow of ISF cleared solutes, that might also include at least soluble Aβ fractions.

Arylsulfonamide derivates, on the other hand, selectively block AQP1 ion conductance without affecting water permeability, providing a pharmacological approach to dissociate the dual functional properties of the channel (Kourghi et al., 2017). Most notably, beyond its role in water transport, AQP1 operates as a cGMP-gated non-selective cation channel implicated in cellular dynamics (Kourghi et al., 2016), with ionic conductance linked to regulation of volume homeostasis. Selective inhibition of its conductance using bumetanide-derived compounds- aquaporin blocker-011 (AqB011), aquaporin blocker-007 (AqB007), aquaporin blocker-006 (AqB006) and aquaporin blocker (AqB001) indicates that ion channel activity, rather than water permeability, underlies AQP1-dependent function (Kourghi et al., 2016). Beyond bumetanide-derived compounds, several other sulfonamide-based molecules have been investigated as potential modulators of AQP1 function. This broader class of compounds includes agents capable of influencing AQP1 activity and has been associated with modulation of β- and γ-secretase pathways (Egbujor, 2024; Smirnoviene and Matulis, 2025), directly linking AQP1-targeted pharmacological modulation to amyloidogenic protein aggregation (Bag et al., 2015; Masand et al., 2018; Dakhlaoui et al., 2023; Nevyhostena et al., 2024; Smirnoviene and Matulis, 2025). AqB013, another bumetanide-derivative, serves as a potent bivalent AQP1 and AQP4 inhibitor, reducing both CSF production and ISF drainage, but with a reported effect of reducing alternative APP processing and chronic neuroinflammation (Migliati et al., 2009; Vella et al., 2015).

To date, the only reported AQP1 facilitator is the arylsulfonamide derivative AqF026, which has been showed to specifically potentiate the channel activity of human AQP1 by more than 20% (Yool et al., 2013). Despite the fact that AqF026 has been showed to enhance the osmotic transport of water across the peritoneal mesothelium, and did not potentiate water transport in Aqp1-null mice, suggesting its specificity, its effect has not been yet evaluated for the brain, nor in an AD animal model.

Altogether, due to the fact that AQP1 modulation show divergent effects on amyloidogenic APP processing and CSF/ISF clearance, it is not very probable that a single direct modulator would be used in AD research beyond mechanistic proof-of-principle studies.

### 8.2 AQP1 knockout and Aβ metabolism and clearance

Genetic ablation of AQP1 disrupts transmembrane water flux and leads to multisystem phenotypes characterized by impaired epithelial fluid secretion, including defective CSF handling (Hua et al., 2019). At the level of the choroid plexus, this results in an approximately fivefold decrease in trans-epithelial water transport (Oshio et al., 2003), and a reduction in CSF production of up to 25% (Oshio et al., 2003), limiting in the end the convective component of interstitial solute movement (Oshio et al., 2003), and potentially diminishing Aβ clearance, thus linking AQP1 deficiency to impaired solute clearance in AD (Municio et al., 2023).

In addition to CSF-dependent clearance mechanisms, AQP1 also regulates amyloidogenic APP processing itself at the molecular level. Reduction of AQP1 expression enhances BACE1-dependent APP processing and facilitates Aβ generation, whereas its presence limits APP–BACE1 interaction without affecting the non-amyloidogenic pathway (Park et al., 2021). Loss of AQP1 enhances Aβ generation in association with increased amyloidogenic APP processing and β- and γ-secretase activity, indicating a regulatory role in amyloid homeostasis (Park et al., 2021). These data support the concept that decreased AQP1 shifts APP processing toward the amyloidogenic pathway, consistent with a modulatory role in Aβ production metabolism (Park et al., 2021).

Moreover, mechanistic insights from single and combined AQP knockout models demonstrate that concurrent loss of AQP1 and AQP4 impairs CSF drainage and ventricular distensibility or compliance, while individual AQP1 or AQP4 knockout does not significantly decrease the CSF drainage capacity (Trillo-Contreras et al., 2019).

### 8.3 AQP1 overexpression and Aβ metabolism and clearance

Although AQP1 is physiologically concentrated at the apical membrane of choroid plexus epithelial cells, where it contributes to CSF formation, its expression pattern appears to change in pathological contexts associated with disturbed water balance and amyloid deposition. In AD, AQP1 immunopositive astrocytes are predominantly localized in close association with Aβ plaques in the motor cortex and hippocampus, indicating a disease-specific redistribution toward plaque-associated regions (Misawa et al., 2008). In contrast, AQP1-expressing astrocytes not associated with amyloid deposition are more frequently observed in non-AD aged brains, supporting a pathology-dependent spatial pattern. Experimental data further show that astrocytes constitutively express AQP1, with expression levels remaining unchanged following exposure to Aβ peptide, but increasing under hypertonic stress conditions. At the molecular level, AQP1 interacts with the C-terminal fragment of APP, specifically through its N-terminal transmembrane domains, suggesting a structural association with amyloidogenic processing intermediates. These findings support a role for astrocytic AQP1 in the local microenvironment of amyloid deposition, linking its upregulation and redistribution to plaque-associated pathological processes in AD (Misawa et al., 2008).

Cortical neurons exhibit elevated AQP1 expression during the early stages of Alzheimer’s disease, a phenomenon also observed in 3xTg-AD and 5xFAD transgenic mouse models, where Aβ and tau pathologies progressively accelerate with age (Park et al., 2021). Experimental data indicate that AQP1 overexpression appears to exert a context-dependent, potentially compensatory role. AQP1 accumulates in vulnerable cortical neurons and redistributes to intracellular compartments associated with β- and γ-secretase activity. Functional overexpression studies demonstrate that increased AQP1 levels reduce amyloidogenic APP processing by disrupting BACE1-mediated cleavage and decreasing Aβ production, without affecting the non-amyloidogenic pathway (Park et al., 2021). Conversely, AQP1 reduction has the opposite effect, and enhances BACE1 activity and Aβ generation, thus supporting a regulatory role in amyloid homeostasis. An inverse relationship between AQP1 expression and Aβ levels further supports its regulatory role in amyloid homeostasis, with AQP1 upregulation representing a stress-induced neuronal response that attenuates Aβ production (Park et al., 2021).

## 9 Conclusion

This comparative overview of AQP4 and AQP1 physiology and modulation models reveals a fundamental division of function within the CNS toward water handling that reveals multiple putative therapeutic implications. Thus, AQP4 operates primarily at the parenchymal–vascular interface, governing bidirectional water exchange, extracellular space dynamics, and glymphatic solute clearance along the peri- and paravascular pathways. In contrast, AQP1 functions at the level of fluid-producing epithelial interfaces, modulating CSF production, osmotic–ionic coupling, and upstream ventricular dynamics.

This distinction reframes CNS fluid regulation as a clearance versus production paradigm, rather than as a homogeneous water buffering system. Genetic evidence indicates that AQP1-driven CSF generation sets boundary conditions for downstream AQP4-dependent clearance pathways, such that perturbations at either level propagate through the entire system. Consequently, therapeutic modulation targeting only parenchymal clearance (AQP4) without accounting for CSF production (AQP1), risks limited efficacy or paradoxical outcomes. Temporal dynamics further differentiate aquaporin function. Acute modulation strategies favor transient suppression of astrocytic water influx, as AQP4 inhibition or mislocalization reduces cytotoxic edema and early mortality. Conversely, chronic modulation strategies require preservation or restoration of polarized AQP4 to sustain long-term clearance of metabolites and aggregated proteins, as evidenced by worsened amyloid burden in anchoring-deficient models despite normal or elevated channel expression. Global modulation of aquaporins disregards cell-type specificity, subcellular localization, and disease stage, increases the likelihood of adverse effects. The observation that mislocalization often produces more severe dysfunction than complete gene deletion underscores the fact that indiscriminate facilitation or inhibition should be considered with caution and only after a complete characterization of their mechanisms of action. These mechanistic insights underline clearly that putative future therapeutic strategies must discriminate between aquaporin channel density, localization, and pre-existing functional background.

Altogether, while the complex regulation and structural diversity of aquaporins challenge the development of highly selective small-molecule modulators, integrating advanced omics, imaging, and computational tools with detailed anatomical and histological data of the peri- and paravascular fluid drainage pathways offers unprecedented opportunities for targeted translation in animal models and future therapeutical avenues.
